# Reduced structural complexity of the right cerebellar cortex in male children with autism spectrum disorder

**DOI:** 10.1371/journal.pone.0196964

**Published:** 2018-07-11

**Authors:** Guihu Zhao, Kirwan Walsh, Jun Long, Weihua Gui, Kristina Denisova

**Affiliations:** 1 School of Information Science and Engineering, Central South University, Changsha, Hunan, P. R. China; 2 Department of Psychiatry, Columbia University College of Physicians and Surgeons, New York, NY, United States of America; 3 Sackler Institute for Psychobiology, Columbia University College of Physicians and Surgeons, New York, NY, United States of America; 4 Division of Developmental Neuroscience, New York State Psychiatric Institute, New York, NY, United States of America; Univdersity Hospital of TübingenUniversitatsklinikum Tubingen, GERMANY

## Abstract

The cerebellum contains 80% of all neurons in the human brain and contributes prominently to implicit learning and predictive processing across motor, sensory, and cognitive domains. As morphological features of the cerebellum in atypically developing individuals remain unexplored in-vivo, this is the first study to use high-resolution 3D fractal analysis to estimate fractal dimension (FD), a measure of structural complexity of an object, of the left and right cerebellar cortex (automatically segmented from Magnetic Resonance Images using FreeSurfer), in male children with Autism Spectrum Disorders (ASD) (N = 20; mean age: 8.8 years old, range: 7.13–10.27) and sex, age, verbal-IQ, and cerebellar volume-matched typically developing (TD) boys (N = 18; mean age: 8.9 years old, range: 6.47–10.52). We focus on an age range within the ‘middle and late childhood’ period of brain development, between 6 and 12 years. A Mann-Whitney *U* test revealed a significant reduction in the FD of the right cerebellar cortex in ASD relative to TD boys (*P* = 0.0063, Bonferroni-corrected), indicating flatter and less regular surface protrusions in ASD relative to TD males. Consistent with the prediction that the cerebellum participates in implicit learning, those ASD boys with a higher (vs. lower) PIQ>VIQ difference showed higher, more normative complexity values, closer to TD children, providing new insight on our understanding of the neurological basis of differences in verbal and performance cognitive abilities that often characterize individuals with ASD.

## Introduction

Our brains are model-makers of the physical world, encoding sensory information in a way that exploits spatial and temporal statistical regularities in the data for efficient representation of, and interaction with, the environment. Strikingly, given the 4:1 ratio of neurons in the cerebellum relative to the cerebral cortex (which contains only 20–25% of all brain neurons [1, 2]), in-vivo morphological computational anatomy neuroimaging investigations that target potential deviations from normal structural and functional architecture of the brain focus primarily on characterizing gray and white matter of the cerebral cortex; morphological features of cerebellar cortex in atypically developing individuals remain unexplored.

Since cerebellar structure is highly irregular and convoluted, a lack of suitable in-vivo imaging analytic computational anatomy techniques has made the study of abnormalities of cerebellar structure challenging. We address this technical challenge by using three-dimensional (3D) fractal analysis of cerebellum extracted from high-resolution magnetic resonance imaging (MRI) scans, a computational approach that actually harnesses the presence of surface irregularities in objects and allows for their quantitative study. The cerebellum has been traditionally considered to be primarily involved in sustaining or supporting motor control. A preponderance of empirical evidence [3], however, which we detail below, suggests a crucial role for the cerebellum in many domains of human cognition and perception (including implicit learning and predictive processing). Further, alterations in cerebellar structure and function may contribute to atypical development [4]. In particular, neonatal cerebellar damage confers a large non-heritable risk (up to 40%) for developing Autism Spectrum Disorders (ASD) later in life [5, 6], revealing the cerebellum’s vulnerability during sensitive periods in neurodevelopment (as the cerebellum undergoes continued post-natal development relative to other brain structures [3]). This background provides an important motivation to more fully characterize the architecture of cerebellum in typical and atypical development.

In healthy, typically developing (TD) individuals, the cerebellum is thought to be involved in constructing internal models of the surrounding environment [7] and one’s position in space, including kinematic states [8–10]. With regard to acquisition and building of internal models of self-movement (for example, in the context of passive motion) the cerebellum serves as a comparator between the “sensory consequences of active self-motion and the sensory feedback” about one’s position in space [10]. The cerebellum is also involved in estimating and predicting sensory events [11], and timing discrimination [12–14]. In particular, the cerebellum is sensitive to violations of temporal predictions, producing a larger response following an unpredictable omission in sequence of stimuli [11].

Subserving the cerebellum’s role in filtering the fidelity of neural operations [15] is a cerebro-cerebellar feedback loop (circuit): the top-down corticopontine-pontocerebellar pathway and the bottom-up cerebellothalamic-thalamocortical pathway [4, 16]. It is still a debated issue whether and how the cerebellum signals violations from predicted events, with the inferior olive and climbing fibers playing an important role in the process. According to some models of (supervised) learning [17] (also consistent with active inference models [18]) the climbing fibers from the inferior olive may convey violations from expected events to the cerebellar Purkinje Cells (located within the middle layer of cerebellar cortex’ gray matter, GM), which send the output in the form of inhibitory signals to the cerebellar deep nuclei, and then to the thalamus and the cerebral cortical areas [19, 20]. Additional major components of the cerebellar cortex (besides Purkinje cells and parallel fibers) include basket, stellate and Golgi cells (as well as climbing fibers from the inferior olive and glial cells, as noted earlier). Our measure of structural complexity (see below) arguably subsumes, and could reflect, any of these diverse components of the cerebellar cortex.

A uniform or homogenous cytoarchitecture of the cerebellum [4, 21] suggests that this structure performs a common computation (Schmahmann’s “transform” [16]) on diverse inputs, including perceptual, cognitive, affective, and sensorimotor. This architecture is thought to support the transformation of “multisensory information to predictive output” [22]. If neuronal processing by the central nervous system (CNS) is affected in part by the “poor”, noisy, or unreliable quality of input contributed by the cerebellum [23]—a structure important for efficiency of sensory processing [24]—then structural alterations of the cerebellum may have important developmental consequences, for example, affecting the integrity of the circuitry in individuals with neurodevelopmental disorders including ASD. Subtle cerebellar abnormalities may affect refinement or filtering of information across diverse domains in individuals with ASD by impacting down-stream systems—for example, via impaired weighting of (sensory) input [25] along the cerebellothalamic-thalamocortical pathway.

### Specific problems that occur following (structural) cerebellar damage

Individuals who sustain non-congenital cerebellar damage may have difficulty estimating temporal deviation of expected events [26], exhibit impairments in appropriate representation of the temporal order of events (“cognitive sequencing” [27] as well as have difficulty in receptive and expressive speech [28]. In a study of children (mean age 8.65 years) undergoing cerebellar tumor surgery, individuals exhibited impairments post-surgery in cognitive ability scores, including verbal intelligence and syntax, as well as behavioral disturbances that were not present prior to the surgery [29]. Further, it has been noted [30] that damage to the left cerebellar hemisphere is associated with impairments in visuospatial processing whereas right hemisphere lesions affect verbal memory and language [31, 32].

### Abnormalities in the cerebellar structure in ASD

Abnormalities in the cerebellar structure in participants with ASD relative to healthy controls, at both the micro- and macro- scale of analysis, have been reported, however, we note that available findings are not entirely consistent across samples studied and within and across diverse analytical approaches, including postmortem/histological and in-vivo MRI studies.

### Postmortem/Histological studies

The majority of histological studies have reported abnormalities in the cytoarchitecture of the cerebellar cortex in individuals with ASD, in particular, by documenting a reduction in the number of Purkinje Cells (PC) [33–36], although one relevant study [37] did not find a consistent difference between ASD and controls’ tissue, with only 3 out of 6 ASD participants showing PC loss.

### MRI

In-vivo, early voxel-based morphometry work reported bilateral increases in GM volume in the cerebellum in participants with autism relative to controls [38]. In contrast, Courchesne and colleagues found reduced cerebellar GM in autistic children (boys), with a smaller ratio of gray to white matter, and smaller vermis lobules VI-VII than normal controls [39]. Further, McAlonan and colleagues [40] found a reduction in cerebellar GM in adult patients with Asperger’s relative to normal controls while a reduction in the white matter (WM) volume of the cerebellum of autistic children relative to controls was reported in a different study by the same group [41]. Akshoomoff and colleagues [42] studied subgroups of participants including those with high and low functioning ASD, and found significant differences in cerebellar WM volume relative to the control group, but no detectable between-group differences in cerebellar GM volume. Recent work by D’Mello et al. [43] reported reduction in GM in the cerebellum in ASD relative to TD children, but regional reductions varied depending on the analytic method (SUIT vs. VBM) [43].

### DTI, fMRI

Neuroimaging studies using Diffusion Tensor Imaging (DTI) [44] and functional MRI (fMRI) modalities [45, 46] further suggest atypical cerebellar structure and function in ASD. Using Diffusion Tensor Imaging (DTI), Jeong and colleagues [44] found atypical connectivity between the right cerebellum and cerebral cortex in sedated children with ASD relative to non-sedated control subjects (N = 15 ASD, N = 14 TD, age range for both groups ~4–14 years) [44]. Further, measuring Blood Oxygenation-Level Dependent (BOLD) signal during an fMRI scan, Wang and colleagues [46] reported significantly reduced activation of the bilateral cerebellum of boys with ASD (N = 18) relative to TD boys (N = 18) (viewing and listening to comments on potentially ironic scenarios), although the between-group differences did not survive after the authors accounted for differences in the lower VIQ ability of ASD participants. On the other hand, Mostofsky and colleagues [45] measured BOLD signal during a motor-tapping fMRI task and found reduced activation of bilateral cerebellum in high-functioning children with ASD (N = 13) relative to TD children participants (N = 13).

Although the results of the above studies generally suggest the presence of atypical features in the cerebellum in individuals with ASD, the overall patterns are inconsistent across studies. Moreover, as suggested in Haar et al. [47], previous findings of significant between-group differences may be due to diverse samples and/or lack of control for important confounding variables, such as brain volume, age and sex. Extreme individual phenotypic heterogeneity of ASD individuals may produce between-group differences in a specific study, but also makes it difficult to replicate a specific finding from one lab across new samples in other labs, at least when using conventional analytic techniques [47]. Alternatively, it may be that important and clinically relevant but subtle perturbations exist but may not be detectable in-vivo using conventional analytic approaches.

### Current study

To the best of our knowledge, no prior work has addressed the question of whether subtle morphological perturbations are detectable in-vivo in the cerebellum in ASD, in the absence of between-group volume differences. Here we examine morphological features of the cerebellar cortex using three-dimensional (3D) fractal geometry approach in male children with a research-reliable diagnosis of ASD relative to TD boys, using a sample characterized by a narrow age range (~7 to 11 years old), normal intelligence quotient (IQ), as well as an absence of cerebellar volume difference between ASD and TD groups. Specifically, we focus on an age range within the ‘middle and late childhood’ period of brain development, between 6 and 12 years, according to Kang and colleagues [48]. We selected the youngest participants with a maximum age approximately before the onset of puberty, normally around 12 years of age for males. We matched participants on Verbal IQ (VIQ) and cerebellar volumes, producing a sample of 20 participants in the ASD cohort and a corresponding, matched TD cohort. (Because of the smaller number of TD children within the targeted age range, fewer matches for ASD participants were available; for additional details on our inclusion and exclusion criteria, see Methods).

**Fig 1:**
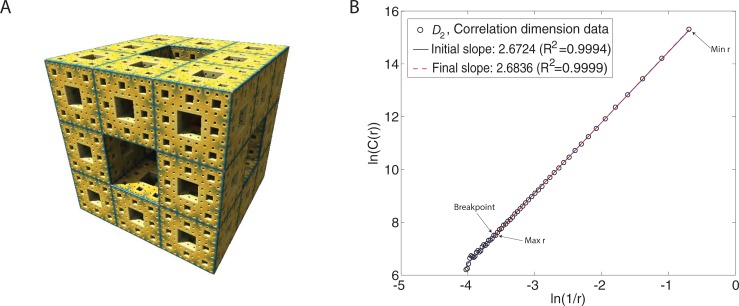
**Validating FD algorithm using the Menger sponge phantom, shown in A; B shows corresponding log-log plots for the *D***_**2**_ **fractal dimension measure. (A)**. Menger sponge 5-iteration phantom is a mathematical fractal made by dividing a cube into 27 smaller cubes and removing seven of the cubes, a process repeated for each of the subsequently smaller cubes 5 times (image size 486 x 486 x 486; the dimension of the smallest hole is 2; thus, the size of each hole is: x = 2, y = 2, z = 2). The expected FD of Menger Sponge is 2.726833. The Menger sponge is rendered using Mandelbulber (https://github.com/buddhi1980/mandelbulber2) open-source software. **(B)**. Correlation dimension, *D*_2_, measure. In the scatterplot of log(*r*) versus $\log(\sum_{i=1}^{Nc(r)}p_{i}^{2})$, *r* is the box size and $\log(\sum_{i=1}^{Nc(r)}p_{i}^{2})$ is the information for box size *r*. *Note*. The initial, pre-determined range of box sizes is *r* = 2… 243 voxels (in increments of 1 voxel). The linear regression analysis is performed iteratively, and the blue line indicates the linear fit over the entire range of *r*. The red dotted line indicates the final fit (*R*^2^); the slope of this line corresponds to the fractal dimension, *D*_2_. The breakpoint separates non-linear data points from the data used in the final regression analysis; ln denotes the natural log. *Min r* is the new smallest box size and *Max r* is the new largest box size.

Our investigation aims to characterize potential differences in the cerebellar morphology, in-vivo, in children with and without ASD using fractal analysis, a fractal geometry technique that captures statistical complexity of an object. Fractal analysis quantifies the structural complexity of an object at successively smaller scales or resolutions. Note that mathematical fractals such as the Menger sponge (**Fig 1**) exhibit self-similarity, meaning that zooming in on the object at increasingly higher resolution will continue to reveal structural aspects similar to those of the larger object, *ad infinitum*. We note that natural objects such as the human brain, exhibit only statistical self-similarity, that is, self-similarity within a limited range of spatial scales [49].

Fractal analysis is an appropriate tool to quantify highly irregular, convoluted features of the cerebellar structure as this technique does not require smoothing of the input prior to analysis, an important consideration allowing capture of subtle and informative surface structure features such as small protrusions (bumps or convexities) and indentations (concavities) (i.e., in contrast to a technique such as a gyrification that quantifies the relation between gyri and sulci but that requires a relatively uniform, smooth surface as input). This is relevant to the current work since potential subtle alterations in the architecture of the cerebellum may reflect a neurobiologically-grounded feature of early vulnerability to aberrant developmental processes in humans. We have previously established empirically that fractal dimension (FD) of an object is (linearly) independent of that object’s volume [49]. This scale-free aspect of FD is important because conventional volumetric techniques need to consider allometric-driven relations between volume and head and/or body size.

We find significantly reduced structural complexity (i.e., FD) of the right cerebellar cortex in boys with ASD relative to TD controls. In ASD, FD is higher in ASD individuals who have a larger PIQ>VIQ difference, relative to those with a lower or narrower PIQ>VIQ spread. We consider these alterations in cerebellar morphology in the context of dissociable learning-based signatures in atypically developing children.

## Materials and methods

### Experimental design

Data were obtained from the open-access Autism Brain Imaging Data Exchange (ABIDE) (http://fcon_1000.projects.nitrc.org/indi/abide/abide_I.html) database. All datasets are de-identified in compliance with U.S. Health Insurance Portability and Accountability Act (HIPPA) guidelines. Research protocols which included neuroimaging and clinical assessments at each site were approved by the local ethics committees. Participants at all sites signed written informed consent and assent (and parental consent, if participants were less than 18 years) in accordance with U.S. 45 CFR 46 and the Declaration of Helsinki for participation. Because the current study did not involve Research with Human Subjects as defined under federal regulations in U.S. 45 CFR 46.101 (b) (4) it is exempt from regulations governing research with human subjects, as per Columbia University Medical Center. We obtained approval and waiver of written/informed consent to conduct analyses on these de-identified data from the Institutional Review Board at Columbia University Medical Center.

### Inclusion criteria

We required potential participants with ASD to have had a research-reliable administration of the ADOS [50] and ADI-R [51], as well as to meet DSM-IV-based clinical diagnosis of ASD. We note that typically developing (TD) child participants at all sites were required to be normally developing, neurologically and psychiatrically healthy individuals, as ascertained via a detailed health questionnaire. The included datasets were from participants in ABIDE who were below 12 years old who were male, and whose verbal IQ (as well as Full Scale and Performance IQ) was greater than 70. Aside from these inclusion criteria, participants with ASD were matched to TD participants on cerebellar volumes and verbal IQ. Individuals with ASD present with variable IQ estimates and often have lower IQ (**S1 Methods)**. To reduce potential sources of variance, in this work we elected to match participants on VIQ.

As ASD is a disorder of development, we wished to understand how brain features differ in ASD relative to TD individuals in childhood, the time of rapid maturation and development. We focused on the age range that corresponds to the ‘middle and late childhood’ period of brain development, between 6 and 12 years, defined according to Kang et al., 2011 [48], selecting the youngest participants approximately up to 12 years (approximately the age before reaching puberty in males). Restricting participants’ age range, at least in cross-sectional research, also reduces potentially confounding variability on brain measures (see below). We restricted the sample to male participants in order to reduce the heterogeneity of participants in the study; genetics studies indicate sex driven differences in ASD (relatively few girls of similar age were available: N = 13_ASD_ and N = 18_TD_).

### Exclusion criteria

We excluded datasets that could potentially produce variability in brain measures. As noted in the Introduction, an individual’s brain volume might be a potential confound in imaging studies. Sex and age (and verbal IQ, of particular relevance for ASD individuals) are additional factors that may interact with one another, affecting brain metrics. Thus, our focus was to quantify differences in the structural properties of the cerebellar GM in the youngest male individuals with a research-reliable diagnosis of ASD relative to those children with typical development, on datasets that both (*i*) passed stringent quality check (QC) and (*ii*) our post-processing examination, while matching on these important variables.

(*i*) Preprocessed Connectomes Project (PCP)-based QC [52]. In the current work, datasets were excluded if raw volume images (T1-weighted) received a rating of “no” or “maybe” from at least one of the two raters in the PCP pipeline. After this initial QC and automatic FreeSurfer segmentation, N = 54_ASD_ and N = 41_TD_ QC-suitable datasets from males below 12 years old were available that had verbal IQ greater than 70.

(*ii*) ASD and TD matching and post-processing segmentations examination. Next, from this sample with usable segmented structural data, N = 54_ASD_ and N = 41_TD_, we chose the 20 youngest male participants with ASD and matched them as closely as possible on VIQ scores and cerebellar volumes (using left and right gray matter volume in mm^3^) to 20 male TD participants of similar age, as follows. Note that as the initial TD male children’s cohort was ~23% smaller than the corresponding ASD cohort, it was not possible to find a unique TD participant for every ASD participant. Importantly, for a given ASD participant, because a smaller pool of TD children were available from which to obtain an acceptable match on the 3 variables of volume, VIQ and age, this constrained the total matches in the final cohorts. We sorted the data such that no between-group differences (*P*>0.05) existed on all three variables, producing final ASD and TD cohorts. Twenty ASD participants were thus matched to 20 TD participants; the remaining participants’ data could not be matched and their segmentations were not examined in the final step. The final post-processing examination step (described below under ‘Preprocessing’ section and in the **S1 Methods**) involved careful examination of automatic FreeSurfer cerebellum segmentations, for each of the 40 datasets. Following this post-processing examination step, two TD datasets were excluded, leaving 18 TD and 20 ASD participants in the study.

We attempted to keep the matches within a given site; however, given our goal to leverage the entire sample and a relatively small number of very young participants even in this large sample (considering the targeted age range for our study), a matching TD participant was sometimes chosen from a different site. Previous work has documented the robustness of FreeSurfer performance on segmentations across different scanner models and image acquisition parameters [53, 54]. Out of the final 38 datasets in the current study, 71% were from NYU (18 ASD, 9 TD). Two additional ASD datasets were from Yale. The rest of TD datasets were from UM_1 (N = 5), UCLA_1 (N = 2), and USM (N = 2) (see **S1 Methods** for additional details).

### Characteristics of the sample

Demographic characteristics of the sample are presented in **Table 1**. Participants did not differ overall with respect to age, 8.8004 years (1.0543) (mean and standard deviation; range: 7.13–10.27) for the ASD group, and 8.9827 years (1.1683) (range: 6.47–10.52) for the TD group (*P* = 0.6162). Out of 38 male children participants, 17 boys with ASD were right-handed and 3 were left-handed; 15 TD boys were right-handed and 2 were left-handed (handedness score was missing for 1 TD participant). Left/Right handedness ratio did not differ between groups: *X*^2^ = 0.0823, *P* = 0.7742. ASD and TD participants did not differ in Full Scale (*P* = 0.13) or Verbal IQ (*P* = 0.66); Performance IQ (PIQ) was higher in ASD compared to TD (ASD PIQ 121.00 +/- 18.83, TD PIQ 104.06 +/- 14.91; mean +/- sd, *t*(36) = 3.05, *P* = 0.0043). In the current study, N = 18 individuals with ASD and N = 12 individuals with TD were assessed with WASI [55]. (Note that WASI [55] is the most common instrument used to estimate IQ in ABIDE; see **S1 Methods** and **S2 Fig** for additional information). ASD and TD participants did not differ (all *P*>0.05) in either right or left GM cerebellar volumes (mean mm^3^ +/- standard error of the mean) (right cerebellar cortex, ASD: 53348 (2100.8) vs. TD: 55826 (1737.7), *P* = 0.3755; left cerebellar cortex, ASD: 54188 (1849.7) vs. TD: 55091 (1646.2), *P* = 0.7199). ASD and TD participants did not differ in total cerebellar cortical volumes, ASD: 107540 (3886.3) vs. TD: 108820 (3498.9), *P* = 0.8076.

**Table 1:** Demographic characteristics of the sample.

	ASD (N = 20)	TD (N = 18)	Analysis (df)	*P*
Age (years)	8.8 +/-1.05	8.98 +/- 1.17	t_(36)_ = -0.51	0.62
Sex, M	20	18		
Handedness^¶^, R/L	17R/3L	15R/2L	*Χ*^*2*^*(1)* = 0.08	0.77
Full Scale IQ	115.15 +/-17.99	107.64 +/-10.36	t_(36)_ = 1.55	0.13
Verbal IQ	108.10 +/- 13.95	110.00 +/-11.98	t_(36)_ = -0.45	0.66
Performance IQ	121.00 +/- 18.83	104.06 +/-14.91	t_(36)_ = 3.05	0.004
ADOS repetitive & restricted behaviors	3.25 +/-1.74	-		
ADOS social affect	8.4 +/- 3.47	-		
ADOS total	11.6 +/- 4.71	-		
ADOS severity	6.65 +/- 2.13	-		
ADI-R^#^ repetitive behaviors	5.68 +/-2.94	-		
ADI-R social	18.37 +/- 5.78	-		
ADI-R communication	15.47 +/- 3.98	-		

ADOS, Autism Diagnostic Observation Schedule; ADI-R, Autistic Diagnostic Instrument-Revised; IQ, Intelligence Quotient.

All ASD and TD participants were required to meet inclusion criteria of Full Scale IQ>70. Means and standard deviations are shown.

¶Handedness score was not available for 1 TD participant.

^#^ADI-R Parent Interview domain scores were available for 19 ASD participants.

In the current version of the DSM-5, diagnoses of ASD and Attention Deficit/Hyperactivity Disorder (ADHD) are not mutually exclusive. That is, an individual may obtain a research-reliable diagnosis of ASD in the presence of ADHD. Twelve ASD participants had a secondary, comorbid neuropsychiatric diagnosis in addition to the primary diagnosis of ASD (these include N = 6 with ADHD subtypes). Individuals with ADHD have difficulties functioning in school settings. This applies across different ‘subtypes’ of ADHD, including “hyperactivity and impulsivity”, “inattention” or those with a “combined” presentation. As such, our analyses on IQ investigated the issue of potential learning atypicalities in the current subset in a *data-driven* manner; we examined individuals based on their PIQ>VIQ spread profile (given matched-to-similar VIQs relative to the TD cohort) in this dataset. We also studied subgroupings of individuals by whether or not they had a secondary diagnosis (comorbidity) in addition to ASD. In future work with larger subsets, it may be possible to stratify ASD individuals by the presence or absence of specific neuropsychiatric comorbidities.

Three ASD participants were on medication: stimulants (N = 1), blood-pressure reducing medication (N = 2), SSRIs (N = 1) (total does not equal 3 because 1 participant was taking more than one medication). Medication status was not available for 2 out of 20 ASD participants. None of the TD participants were taking medication at the time of the scan.

### MRI acquisition parameters

High-resolution structural images were obtained using T1-weighted pulse sequences at 3T MR scanners at all sites, on Tim Trio at UCLA_1, USM, and Yale and on Allegra (Siemens, Erlangen, Germany) at NYU, and on a Signa (GE Medical Systems, Milwaukee, WI) at UM_1.

Additional information on site-specific ABIDE inclusion/exclusion criteria, recruitment information, and image acquisition parameters are listed in the **S1 Methods**.

### Preprocessing

Cerebellum 3D volume images were obtained using the Preprocessed Connectomes Project (PCP) resource [52], which utilized default anatomical image workflow in FreeSurfer to preprocess raw T1-weighted images in ABIDE, with resulting segmentations denoted as “aseg” data files. FreeSurfer has automatic and semi-automatic (which allows user intervention) pre-processing pipelines; we opted for the automatically pre-processed segmentations, which we then inspected for accuracy. FreeSurfer version 5.1 (http://surfer.nmr.mgh.harvard.edu) was used by PCP to process raw volume images and for cerebellum reconstruction. No user intervention was applied during automated pipeline processing (‘recon-all–i data1.dcm–all’). The pipeline for the recon-all command consists of several major steps: (*i*) intensity normalization, (*ii*) removal of non-brain tissue (“skull-stripping”), (*iii*), further normalization, based on the Gaussian Classifier Array (GCA) model, and (*iv*), labeling of cortical and subcortical regions. Briefly, the intensity normalization is based on computing the affine transform from the original volume to the MNI305 atlas using the Avi Snyders 4dfp suite of image registration tools. In the intensity normalization step, FreeSurfer attempts to correct the fluctuations in intensity of the original volume; this correction facilitates the intensity-based segmentation. The skull-stripping step removes the skull from the image. Following the skull-stripping there is a further normalization step used to estimate the bias field, based on the GCA model (note that GCA assigns voxel information probabilistically using a training set). After obtaining the final normalized image, the routine labels subcortical structures, again based on the GCA model, and generates the aseg.mgz files for brain structures. Additional details of FreeSurfer segmentation are described in Fischl et al. [56].

Using FreeSurfer 5.1 running under Ubuntu OS, we examined for accuracy these automatic segmentations (3D volume images denoted as ‘aseg’ files) for left and right cerebellar cortex, working primarily in the sagittal plane, against a corresponding T1-weighted image for each participant, and corrected when necessary (see additional details on the examination procedure in **S1 Methods**). Note that with regard to aseg boundaries, we can confidently exclude potential errors made by FreeSurfer as contributing for the final GM boundary because we carefully monitored for such errors at the “outer” as well as the “inside” boundary. That is, no WM is part of GM. However, by virtue of the figure/ground relation for the “inside” boundary, some of the GM boundary is shared by WM as well; the contour’s shape *per se* may be due to WM parameters impinging on GM or rather GM impinging on WM. Given a biological object, potential differences in protrusions (vs. indentations) in the ASD relative to the TD group would represent contributions of both the outer boundary as well as the boundary that was “inside” (i.e., the shared contour representing WM and GM boundary).

Of course, given the natural continuity of the underlying brain tissue, distinguishing between “GM” and “WM” is a challenging conceptual question. Contrary to most other structures (e.g., the cerebral cortex) in the central nervous system, the cerebellum (cerebellar peduncles, as well as the inferior olive) is one of the few structures (after brainstem) to show microscopic myelin at birth [57], and undergoes protracted maturation in childhood. In general, though, increases in age are accompanied by increases of myelin and protein as well as decreases in water content in the brain and give rise to well-defined contrast between different tissue types on MR images. Because by toddlerhood, as T1-weighted images already have an adult-like appearance, with gray matter appearing darker and white matter appearing lighter, tissue intensity can be used to distinguish the two tissue types in children within the age range studied here. This property is what makes it possible for humans and machines (including algorithms in FreeSurfer) to rely on differences in tissue intensity to segment image into GM and WM. Briefly, in the context of the cerebellum, FreeSurfer considers as WM, the deep nuclei as well as thinner branches that extend into GM. The left and right hemisphere assignment in FreeSurfer proceeds across the midline, meaning that hemisphere designations are split across the vermis; it does not assign a separate label to the vermis. Currently, FreeSurfer does not segment cerebellar lobules. In summary, the same software, FreeSurfer, which we have found preferable to other environments (e.g., 3D Slicer) for this purpose, was used in our examination of the automatic segmentations against the original, high-resolution T1-weighted MR image for each participant. Since we were examining global properties of the cerebellum, our examination scheme is considered “coarse parcellation” according to Schmahmann [58] terminology. Our protocol follows an abridged version of Bogovic et al. [59] protocol, described in detail in **S1 Methods**.

To prepare image files as inputs for fractal analysis (described in detail below), we converted the 3D object to a 3D binary image. We then centered the 3D volume image by removing the blank space in the image and zero-padding it in x, y, and z directions (the sequence of the image processing pipeline is illustrated in the flowchart in **S1 Fig**). In addition, we used FreeSurfer routines to compute volume (mm^3^) for each left and right cerebellar cortex, for each participant.

### Analytics: Fractal analysis

We estimate fractal dimension (FD) using an established and validated probabilistic distance-sensitive metric based on the box-counting approach. We first note that box-counting is (*i*) appropriate for evaluating objects with and without strict self-similarity, including natural objects such as the human brain, (*ii*) suitable for measuring dimensions of sets of points or volume elements [60, 61], and (*iii*) has been validated for optimization and reproducibility using two imaging datasets [62].

Let us first define a *set* to be a three dimensional (3D) right and left cerebellum gray matter (GM) structure formed by voxels. Fractal analysis proceeds by first covering the set with a 3D grid of differently sized cubes of size *r* (hence the distinct sizes of cubes of different 3D grids represent the different scales or resolutions applied to the set). For a given set, “dimension” represents a scaling exponent of the set with its size (across different scales or cube sizes): *set* ∝ *size^Dimension^*. This power-law relation states that the frequency of finding a portion (e.g., a protrusion) of the object of a given size is proportional to the set’s size. The relation between cube size *r* and dimension is inversely proportional: *set* ∝ *r^−D^*.

In the current work we utilize *D*_2_, the correlation dimension, an elegant distance-sensitive representation of dimension. *D*_2_ is implemented using a probabilistic framework, meaning that it accounts for possible inhomogeneities in the set by representing whether the cube contains few or many voxels in the set, and it provides “an estimate of dimension based purely on the statistics of pairwise distances” [63].

Below we define several terms as follows. Specifically, *p_i_* is the probability of finding a voxel of the fractal object falling into (or intersecting with) the *i*-th box in a 3D grid of side *r* as a function of a total number of voxels (*N_all_*): *p_i_* = *N_i_*(*r*)/*N_all_*. *N_i_*(*r*) denotes the number of voxels which are covered by the *i*-th cube in a 3D grid of regular sides of length *r* (in increments of 1). *N_all_* is the number of voxels of the 3D fractal object.

Thus, the correlation dimension is expressed as:

$$D_{2}=\underset{r\to 0}{\lim}\frac{\log(\sum_{i=1}^{Nc(r)}p_{i}^{2})}{\log(r)}$$ (1)

Letting $C(r)=\sum_{i=1}^{Nc(r)}p_{i}^{2}$, we obtain

$$D_{2}=\underset{r\to 0}{\lim}\frac{\log C(r)}{\log(r)}$$ (2)

As noted in Grassberger [64], *C*(*r*) can be defined using 2^nd^ order Rényi entropy [64–66]: $C(r)=\sum_{i=1}^{Nc(r)}p_{i}^{2}$ [64]. Intuitively, it means that the statistics of pairwise distances is approximately equal to the probability that “two points of the set are in the same” cube [67]. The stability and reproducibility of *D*_2_ when expressed by *p_i_* using box-counting has been established in previous work [62].

We follow our previously published computational procedures for computing FD from MR images [49], which we extend here to the study of the cerebellum. We describe below important parameter specifications that are required for accurate FD computation in general, focusing on several that are specific to the current work, namely (*i*) the choice of box range, (*ii*) the initial parameters, and (*iii*) FD estimation scheme.

The selection of optimal box size (*r*) range (minimum and maximum) is important in practical computations involving physical objects as suboptimal parameters may adversely affect FD computation [61]. Note that while equations above specify a lower *r* size at 0, lim*r*→0 is not attainable [63] when computing FD of real physical (solid) objects due to finite image resolution that restricts possible lower bound. The minimum box size (*Min r*) is 2 voxels [49, 68] while the maximum box size (*Max r*) is equal to the 70% of the shortest side of the image for each cerebellum structure [61] yielding 3D grids of side length in the range of *r* = 2 to 70% of the smallest Euclidean dimension of the object. Note that the use of % yields slightly different maximum values, with the median value approximately 60 voxels and accommodates subtle subject-specific differences in image size dimensions of the cerebellums of different participants.

In addition, the offset of the 3D grid with respect to the “edge” of the object (cerebellar structure) within an image may affect the value of fractal dimension [62, 69–71]. For example, the FD estimates may be slightly different depending on the choice of the offset position. In current work, we apply 20 randomly positioned offsets of the 3D grid (for each size *r*, in the range of min-max above) in order to reduce systematic bias [62], and use the median value for a given *r* size for subsequent computations. In this work, our computational routine starts with the minimum box size (2) and iteratively proceeds to increasingly larger box sizes, up to the maximum box value (70% of the smallest dimension of the image for each cerebellum structure), in increments of 1 voxel. FD value is obtained by iterative linear regression analysis [49], as follows.

Specifically, the log-log plots are initially plotted using the entire range of box sizes that have been determined *a priori* as described above, and the initial FD estimate is computed as the slope of the log-log regression line. Note that the points that deviate from the line of regression fit in the log-log plot appear as a non-linear distortion in **Fig 1B**. Because such points do not provide further complexity information about an object, regression fits are repeated, by systematically excluding these points, until a straight line that includes most data points can be fitted [61]; the final fit is chosen so that it produces the largest *R*^2^. The point that distinguishes the linear and non-linear parts of the data is called the “breakpoint” (indicated by the arrow in **Fig 1B**). We find that there were systematic differences between the ASD and TD group in the breakpoint above which the data were judged linear for both right and left cerebellums (**S1 Table**).

The data points utilized for the final linear regression fit include the revised upper bound (i.e., largest box size, Max *r*) and the lower bound (i.e., the smallest box size, Min *r*); see **Fig 1B**. In the final step, the Fractal Dimension (FD) measure is obtained by fitting a linear regression (least squares) on the scatter plot of log(1/*r*) versus log(*D*_*2*_(*r*)). Thus, the slope of this final regression corresponds to the FD estimate (**Fig 1B**). We perform these steps for left and right cerebellar cortex and apply Bonferroni correction to guard against multiple comparisons when comparing ranked FD median values for the two diagnostic groups (please see Statistical analyses section). We further note that the individual fits were highly linear: the coefficient of determination (*R*^2^) for the linear regression model fits to the data were as follows: *D*_2_: right cerebellum > 0.996, and left cerebellum > 0.995.

We validated our algorithm using a 5-iteration Menger sponge (dimension of the smallest hole set to 2) constructed using open-source Mandelbulber software (https://github.com/buddhi1980/mandelbulber2) (image size 486 x 486 x 486) (**Fig 1A**). The dimensions were chosen to produce image size so that it subsumes the largest extent (in either x, y, or z direction) in these cerebellum 3D image data. Our empirical FD estimate was close (2.6859) to the expected or theoretical FD of Menger Sponge ~ 2.726833.

The algorithm was implemented using routines written in MATLAB 8.3 (R2014a). In summary, the routine directly reads 3D binary image file, counts the cubes that contain voxels of the object and constructs log-log relations according to the correlation dimension method for each 3D grid with cube length r, computes slopes in a recursive manner, and determines the final slope value (FD) based on the straight portion of the line. We computed FD for GM of right and left cerebellar cortex, for male children with ASD and typically developing healthy age-matched controls.

### Statistical analyses

A non-parametric Mann-Whitney *U* test (two-tailed) assessed whether structural complexity characterized by the fractal dimension differed between individuals with ASD relative to TD controls (Kolmogorov-Smirnov test determined that data are not normally distributed; **S2 Table**). Bonferroni correction was used to guard against multiple comparisons (0.05/2 = 0.025). Cohen’s U3 effect size (U3, a non-parametric measure of effect size that makes no assumptions about underlying data distributions) was computed. U3 measures the proportion of data points in the lower group (here, the ASD children) that are lower relative to the median of the higher (typically developing) group [72], reported with 95% Confidence Intervals (CIs).

Significant between-group results were further evaluated by testing for the link between cognitive profiles and FD outcome measures. While we strove to ensure that our ASD and TD participants did not differ on Verbal IQ (VIQ) by matching the two groups on VIQ, and the two groups were statistically indistinguishable on Full Scale IQ (FIQ), ASD participants had higher scores on the Performance IQ (PIQ), congruent with previous reports of uneven cognitive profiles in ASD (see **S1 Methods, S2 and S3 Figs**).

We used a non-parametric rank-order Spearman (*rho*) test to explore whether increasingly higher distance between PIQ (relative to VIQ) in ASD is associated with higher structural complexity, FD, reporting partial correlations controlling for age. The corresponding hypothesis for RMSE (root mean square errors to the log-log line of best fit) is that increasingly greater distance between PIQ (relative to VIQ) in ASD is associated with lower RMSE values, also reporting partial correlations controlling for age (SPSS; IBM SPSS 23.0, Armonk, NY: IBM Corp.). Complementary analyses included tests for an age by diagnosis, a site by diagnoses as well as IQ by diagnosis interactions, and also for an association between FD and autism symptoms on the ADOS and FD and presence or absence of secondary diagnoses in ASD (**S1 Results**). Statistical analyses were performed (except as noted) using functions in Statistics and Machine Learning Toolbox or Effect Size Toolbox (for computing Cohen’s U3 effect size [73]), running under MATLAB.

## Results

**Fig 2:**
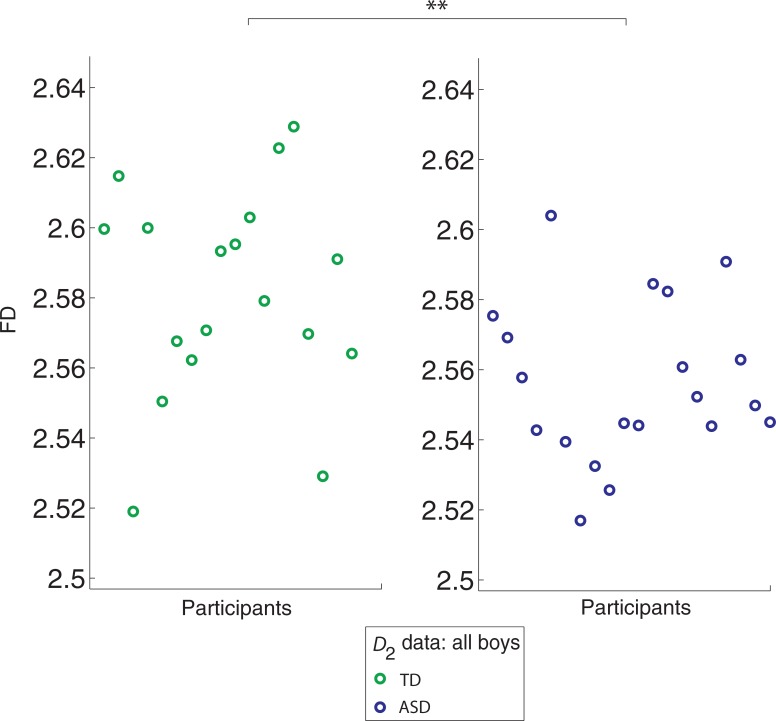
The scatter plot shows individual fractal dimension (FD) values for the right cerebellar cortex. The left panel shows FD values for typically developing (TD) children (N = 18) and the right panel FD for ASD children (N = 20). **denotes *P*<0.05, Bonferroni correction.

A Mann-Whitney *U* test indicated a significant reduction in FD in the right cerebellar cortex in ASD relative to TD individuals that survived a Bonferroni correction (ASD median: 2.5511, range: 2.5170–2.6040 vs. TD median: 2.5851, range: 2.5191–2.6289, *U* = 86, *P* = 0.0063) (**Fig 2**). Cohen’s U3 effect size of the FD difference between groups was large [73], indicating a minimal overlap between FD values of ASD and TD children for the right cerebellar GM (*D*_2_, U3 = 0.9 (95% CIs: 0.6, 1). Although highly linear, the final regression fits, *R*^2^, showed a significant reduction in linearity in individuals with ASD (mean +/- std): ASD, 0.9969±0.000118, TD, 0.9976±0.000169, *P* = 0.0015. In addition, we detected significantly lower root mean square error (RMSE) values in the TD group, indicating a better fit for TD children. The RMSE values (mean +/- std) were, for the right cerebellum cortex: ASD: 0.1076±0.00206, TD: 0.09367±0.00334, *P* = 0.0009.

**Fig 3:**
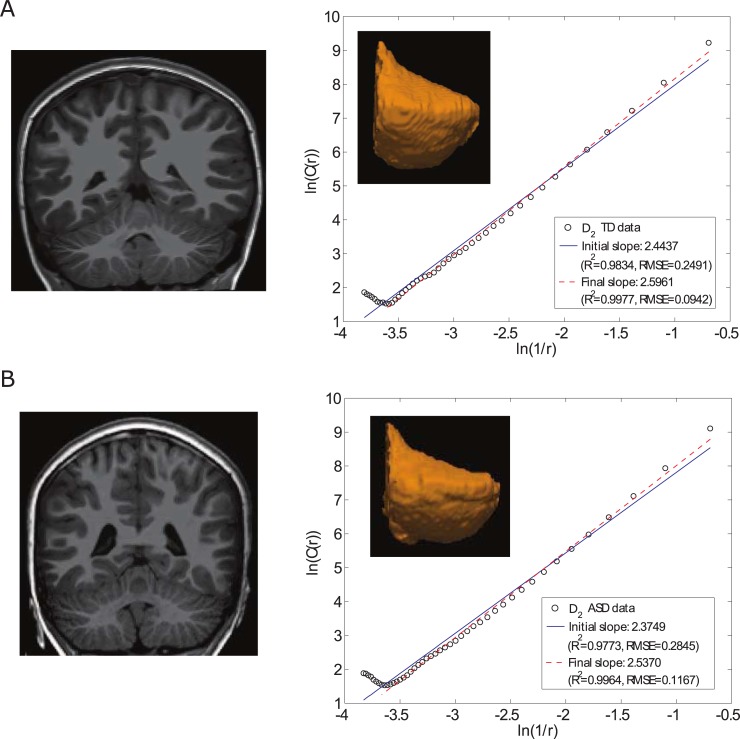
**Illustration of subtle surface non-linearities in the right cerebellar cortex of two individual participants (one TD and one ASD) as captured by the FD measure (*D***_**2**_**), (A)** TD male child (9.21 years old, UCLA 0051278 in ABIDE) and **(B)** ASD male child (10 years old, Yale 0050602 in ABIDE. The left panel shows the bilateral cerebellums in the coronal plane whereas the right panel shows a rendering of the right cerebellar cortex for each participant and its corresponding log-log plot. Note that the final slope estimate (i.e., the FD value) is higher for the TD participant, with higher *R*^2^ and lower root mean square error (RMSE), indicating a better fit for TD individual relative to the participant with ASD.

The reduction in *R*^2^ (as well as the higher errors to the fits) in the right cerebellar cortex for the ASD group indicates that across different boxes of size r, the *C*(r) estimates (y-axis in **Fig 3**, shown for two representative participants) do not increase linearly (monotonically) with increasing box-size increments in ASD relative to TD, who show more uniform increases of *C*(r) estimates across increments, a subtle trend reflected in significant (*P* = 0.0015) between-group differences in *R*^2^. No FD difference was detected in the left cerebellar cortex between ASD and TD participants (*P*>0.05) (**S3 Table**).

While ASD and TD individuals did not differ in the proportion of right-handed vs. left-handed individuals **(Table 1**), we ascertained that the main findings hold by analyzing data obtained only from right-handed individuals. To rule out the possible influence of subjects who were left-handed on FD reduction, we repeated main analyses on participants who were right-handed (N = 17_ASD_, N = 15_TD_). This validation analysis confirmed our main findings of significantly reduced FD in the right GM cerebellar cortex (*P* = 0.008) and higher RMSE (*P* = 0.0051) in ASD relative to TD individuals (**S1 Results**).

### Exploratory analyses: FD and PIQ>VIQ profile in ASD

We next explored whether FD values in the right cerebellar cortex varied as a function of boys’ cognitive abilities. ASD individuals often have lower verbal ability scores relative to their non-verbal scores (**S1 Methods; S2 Fig**), and in males with ASD, this phenotypic feature may underlie differences in their underlying genome [74]. It was therefore important to keep one of the scores fixed in order to limit potential sources of variability in brain surface morphology; thus we elected to include ASD and TD male children with equivalent Verbal Intelligence Quotient (VIQ) scores (*P*>0.05). In addition, individuals with ASD often have variable cognitive abilities, such that PIQ may be higher relative to VIQ, as well as that the relative distance between the two subscores is wider relative to TD individuals. This pattern is present in the overall ABIDE sample (**S1 Methods; S2 Fig**) and in our current subset (**S3 Fig)**, and may be accentuated when IQ in atypically developing populations is assessed using Wechsler-based instruments [75, 76] (**S2 Fig**).

First, because there is a wide range of Full Scale IQs (FIQ) (i.e., standard deviation: 17.99 in ASD participants), it was important to investigate whether there is an association between FIQ and FD, and whether this association is the same in both groups. In this dataset, we did not detect a statistically significant difference in FD between those with lower vs. higher FIQs, either when using the entire children’s sample or examining each diagnostic group separately (all *P*>0.05, **S1 Results**). We next examined the association between Performance IQ (PIQ) scores and FD. No significant association was detected, either when using the entire children’s sample or examining each diagnostic group separately (all *P*>0.05, **S1 Results**). In addition, when considering VIQ scores and FD, no significant association was detected, neither when using the entire children’s sample nor when examining each diagnostic group separately (all *P*>0.05, **S1 Results)**. Further, we examined the association between PIQ-VIQ and complexity. In the TD group, no significant associations were detected. Specifically, the Spearman test (partial correlation correcting for age) showed a positive relationship between higher PIQ>VIQ and FD: *r*_*s*_(15) = .218, *P* = 0.2. We observed a negative relationship between higher PIQ>VIQ spread and RMSE: *r*_*s*_(15) = -.269, *P* = 0.148. This outcome is not surprising, as the TD group was comprised of individuals some of whom had higher scores on their verbal component of the IQ relative to the performance component (VIQ>PIQ), and others with a reverse pattern of PIQ>VIQ (TD N = 12 had VIQ>PIQ, TD N = 6 had PIQ>VIQ) (**S3 Fig**).

We posit that individuals whose PIQ score is higher than their VIQ score have a distinct different cognitive profile compared to individuals with a reverse profile (i.e., those whose VIQ scores are higher than their PIQ scores). In order to reduce variability in the analysis and because the majority of the ASD sample had scores such that PIQ>VIQ, we next studied 17 of 20 (85%) ASD boys with higher PIQ relative to their VIQ score. Thus, PIQ>VIQ is always positive, but the distance between the two scores could be larger or smaller.

**Fig 4:**
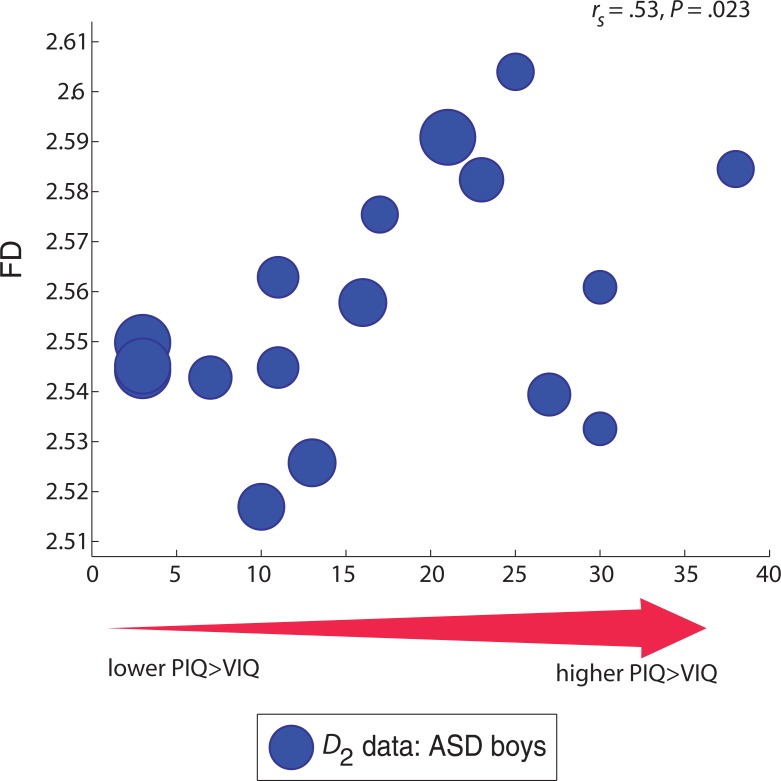
The scatter plot showing the association between individual FD values and PIQ>VIQ profiles for ASD boys. Data shown are from ASD participants with higher PIQs relative to their VIQ scores (N = 17 out of 20 ASD participants have PIQ>VIQ scores). Overall FD is *higher* for ASD participants who have a *higher*, wider PIQ>VIQ spread compared to ASD participants with a *lower* or narrower PIQ>VIQ difference (*P* = 0.023). The size of the marker denotes the corresponding root mean square error (RMSE) value to the log-log line of best fit for each participant; a smaller marker indicates a lower error value to the line of best fit and a larger marker indicates a higher value. *Note*. PIQ: Performance Intelligence Quotient; VIQ: Verbal Intelligence Quotient. All IQ subtest scores are within the normal range, above 70.

A non-parametric rank-order Spearman test (pa rtial correlation correcting for age) showed a significant positive relationship between a higher PIQ>VIQ spread and FD in ASD: *r*_*s*_(14) = .504, *P* = 0.023, indicating that a PIQ>VIQ profile accounted for approximately 25% of the variance in FD (**Fig 4**). We also observed a significant negative relationship (as expected) between a higher PIQ>VIQ spread and RMSE: *r*_*s*_(14) = -.469, *P* = 0.033, indicating that a PIQ>VIQ profile accounted for approximately 23% of the variance in error to the line of best fit (The size of the symbols in **Fig 4** denotes corresponding RMSE values for each participant; note that larger symbols with larger RMSE values tend to correspond to FD data points located towards the bottom-left portion of the plot). Note that this trend held but was marginally not significant when we included all 20 ASD children in the analysis, including 2 with VIQ scores higher than their PIQ scores, and 1 whose VIQ and PIQ scores were identical. Specifically, the Spearman test (partial correlation correcting for age) showed a positive relationship between higher PIQ>VIQ and FD in ASD: *r*_*s*_(17) = .369, *P* = 0.06. We observed a negative relationship between higher PIQ>VIQ spread and RMSE: *r*_*s*_(17) = -.385, *P* = 0.052. We did not detect a significant correlation (all *P*>0.05) between PIQ-VIQ spread and FD of the *left* cerebellum, either when considering those ASD boys with positive PIQ>VIQ profiles, or the entire ASD cohort.

In addition, we explored the association between scores on clinical assessments in the ASD group and FD (and the corresponding RMSE) values in the right cerebellum. We detected a trend for lower FD in the right cerebellar cortex in those ASD participants with worse (i.e., higher) vs. better (i.e., lower) scores on the Social Affect (SA) behaviors on the ADOS (*P* = 0.07, **S1 Results**). No other associations were significant.

### Complementary analyses

We have limited our cross-sectional subset to a relatively narrow age range in order to reduce potential variability in the sample. However, because participants are aged ~7 to 11 years old, an age range at which structural changes are still important, we probed potential effect of age on FD. No significant difference was detected between the age and structural complexity in this dataset, either when using the entire children’s sample or examining each diagnostic group separately (all *P*>0.05, **S1 Results**). In addition, no significant interaction emerged between different ABIDE sites and group diagnostic status on FD (all *P*>0.05, **S1 Results**).

## Discussion

We show that male children with ASD have significantly reduced structural complexity of the right cerebellar cortex relative to age-, VIQ- and cerebellar volume-matched typically developing 7–11 year old boys. Within the ASD group, Fractal Dimension (FD) is significantly lower for ASD children with a lower, narrower PIQ>VIQ range relative to those with a wider PIQ>VIQ range. Those individuals with ASD who have a higher PIQ (relative to their VIQ) in this sample have increased structural complexity of the right cerebral cortex; that is, more normative structural features with higher FD values closer to those of TD controls. We next consider how these macro-level structural differences may further our understanding of ASD individuals’ atypical abilities in implicit learning and, more generally, how these may adversely affect construction of the internal models of the world needed for efficient interaction with the environment.

### Implications of reduced FD in ASD

Reduction in FD means that fewer differently sized boxes across sampled scales (resolutions) were detected in the ASD group. In the absence of between-group volume differences, reduced structural complexity suggests macro-scale (millimeter-level) surface features that are *flatter* in ASD relative to the TD group (**Fig 3A and 3B** insets). Further, individual log-log fits were more accurate (lower error to the fit) for TD relative to ASD individuals (**Fig 3A and 3B**); this pattern indicates that box counts increase in a more gradual fashion (more linearly) with the corresponding increases in the size of surface protrusions in TD relative to ASD children.

In the absence of histopathological correlation, *in-vivo* neuroimaging studies including ours are unable to link atypicalities in structural complexity to specific cell types and functions. However, because we took extreme care to match cohorts on volume, our findings speak to subtle structural differences in tissue that composes these GM volumes. Our findings of flatter features of the cerebellar cortex, for example, may potentially point to differently-arranged cell bodies or cell parameters (dendrites or axons) of inhibitory (GABAergic) Purkinje cells (PC) and/or stellate, basket, and Golgi cells (i.e., as these types of cells and the accompanying cell body structures constitute GM tissues of the cerebellum) [77]. Subtle structural alterations in the cerebellar cortex may have (downstream) functional consequences, for example, with regard to precision [21] with which cerebellar output is conveyed downstream to other brain structures such as the cerebral cortex. As noted in Fatemi et al. [21], parallel fibers and PC cells are configured to “form a network that specifically detects precisely timed sequences of input activity and generates precisely timed output activity in response” [21], with axons of PC cells projecting to deep cerebellar nuclei, which in turn project to motor, autonomic, and limbic cerebral structures [21]. In turn, reduced precision of cerebellar signals may be associated with increased noise levels, or uncertainty, across levels of the cortical (and subcortical) hierarchy in ASD [78].

This possibility is consistent with recent empirical evidence documenting atypically increased signal variability in spontaneous and goal-directed behaviors in individuals with ASD (relative to TD controls) [78, 79] and in infants at high risk (HR) vs. low risk (LR) for developing ASD later in life [80]). For example, we recently found that HR 1–2 month old infants’ head movements were insensitive to evolutionarily-important differences in sensory information. Specifically, HR infants had abnormally similar head movements while listening to native language compared to when sleeping, whereas age-matched LR infants had significantly noisier head movements while listening to language vs. sleeping. Early differences in perception, including timing, could shape cerebellar development and account for our structural findings in boys with ASD, a possibility that we are currently pursuing.

It is worth noting that the cerebellar cortex was found, by Willsey and colleagues, to be significantly enriched for probable ASD genes in the time period immediately preceding the one studied here, prior to 6 years of age (‘early childhood’, between 1 and 6 years of age) [81]; the authors did not detect enrichment for ‘middle and late childhood’ period. Further, the cerebellum has the “most distinctive transcriptional profile” relative to other regions (including the cortex, hippocampus, and amygdala) [48]. Considering general trends across the time period in our study (between 6 and 12 years), the cerebellum shows a steep increase in genes expressing synapse development, dendrite development, as well as myelination, in surprising contrast to other structures such as the cortex and hippocampus, that show relatively flat levels of expression of these genes [48]. We return to this pattern, implicating early atypical perceptual sensitivity in atypically developing individuals, later in the Discussion.

### Lateralization: FD reduced in the right cerebellar hemisphere in ASD vs. TD

We found that FD is reduced in the right cerebellar cortex in ASD relative to TD individuals; no between-group difference was detected for the left cerebellar cortex. Considering this finding with regard to potential hemispheric lateralization-driven differences in learning between ASD and TD groups, we first note that language processing in TD individuals is normally subserved by the right cerebellar hemisphere (i.e., the hemisphere contralateral to the cerebral cortex’ left hemispheric specialization for right-handed individuals). In particular, damage to right posterior cerebellum is associated with deficits with receptive and expressive language [82].

It is unclear if language hemispheric lateralization is well-defined in ASD, even for right-handed individuals [43]. As such, we note that in our study, there were no differences in either handedness (often used as a proxy for language lateralization) or verbal ability (VIQ) between ASD and TD boys (**Table 1**). The pattern of atypical cerebellar hemispheric activation in ASD appears early in development. For example, early work has shown that while TD toddlers recruited the right cerebellar hemisphere more than the left, as expected during a language task, the activation pattern in ASD toddlers was reversed [83]. With regard to (functional) lateralization, it has been suggested that weak lateralization may be a consequence of atypical language learning [84].

In-utero fetal imaging suggests prenatal anatomic hemispheric asymmetry differences [85, 86], including, for example, earlier development of the right cerebral hemisphere (superior temporal sulcus) than the left, and a larger left temporal lobe relative to the right (i.e., in 2/3rds of fetuses studied [85]). Nevertheless, the origins of language lateralization are debated, as some suggest that functional lateralization may reflect an interaction between heritability and the capacity to learn [84, 87].

Given that atypical neuroanatomical findings in ASD “are often right-lateralized” [43]), atypical hemisphere-specific structural differences in ASD vs. TD male children may be considered an emergent result contributed in part by atypical learning process during early life. Taking these caveats into consideration, a conventional language account may not fully explain our current findings of observed right-lateralized structural differences between ASD and TD children.

Instead, given that the cerebellum undergoes protracted development after birth [3] and therefore may be more vulnerable to atypical post-natal processes impacting cognitive development, including interactions between genetically-driven, environmental, and experiential factors, our current findings in children may be consistent with a mechanism representing an interaction between an early atypical maturational process and an atypical active learning process (consistent with Bishop’s 2013 neuroplasticity model [84]). We next consider this possibility in the context of the FD reduction as a function of PIQ>VIQ profile in the ASD cohort.

### Lower FD for ASD boys with lower PIQ>VIQ spread (vs. higher PIQ>VIQ)

An uneven cognitive profile, such that PIQ>VIQ that often characterizes individuals with ASD, is present in the ABIDE sample (**S2 Fig)** as well as our subset (**S3 Fig**). We found that FD was significantly lower for those ASD children with a lower PIQ>VIQ range relative to those ASD children with a wider PIQ>VIQ range (**Fig 4**). Specifically, ASD children with a wider PIQ>VIQ difference had higher FD (closer to TD children) while ASD children with a narrower range have lower FD (further away from the TD group). Thus the presence of higher PIQs relative to VIQs, reveals more normative structural features of the right cerebellar cortex in ASD individuals—that is, FD values closer to those of TD controls.

Performance IQ subtests measure non-verbal ability. For example, some (e.g., Block Design; WASI [55]) may require timed responses during the precise arrangement of component pieces in accordance with a set goal. Relatively normative cerebellar morphology found in those ASD participants with overall higher PIQs (vs. VIQs) could reflect the contribution of the cerebellum in supporting the integration of motor and cognitive processes. Alternatively, and as suggested above, reduced FD in those with lower PIQ>VIQ could reflect an interaction between impaired maturation of the cerebellum and atypical implicit learning in this subset of ASD male children; this could in turn lead to atypical structural features of the cerebellum and hence contribute to the observed overall differences between the two diagnostic groups.

Verbal IQ captures verbal “ability” by measuring primarily declarative knowledge; however, it does not necessarily reveal one’s competence with respect to phonology or grammar. Further, it does not completely explain the emergence of atypical language competence in ASD *per se* which entails, in part, an implicit learning process soon after birth. The neurobiology underpinning the development of those children who receive ASD diagnoses in the presence of a relatively intact implicit learning ability in middle and late childhood and lower declarative verbal ability or capacity is an important outstanding question for future work.

Given a similar level of VIQ in ASD relative to TD children in our study, facilitated levels of PIQ (vs. VIQ) in ASD boys are associated with higher complexity of their right cerebellar GM structure (similar VIQ and PIQ subtest scores suggests that declarative and (procedural) implicit learning processes are at comparable levels). Therefore, a larger PIQ>VIQ profile may “rescue” GM cerebellar structure in some children with ASD, or alternatively, a higher GM cerebellar complexity may give rise to increased levels of competence in implicit learning.

Our findings further speak to recent work in genetics by Iossifov and colleagues [88] suggesting a multi-class model of familial risk for ASD, particularly, for males diagnosed with ASD who have *lower* non-verbal IQs vs. those who have *higher* non-verbal IQs. Specifically, Iossifov and colleagues reported reduced numbers of *de novo* mutations, as well as non-overlapping distributions of *de novo* mutations for ASD males with higher non-verbal IQ relative to ASD males with lower IQ (as well as relative to unaffected individuals and females with ASD), suggesting distinct, genetically-driven mechanisms underlying differences in cognitive ability profiles in those males with ASD [74, 89]. This line of work showed that the majority of the *de novo* mutations may be targets of the fragile X mental retardation protein (FMRP)-associated genes, thought to contribute to reduced synapse formation and atypical cognitive abilities [88, 90, 91]. Although the contribution of *de novo* mutations to overall autism risk is not large (at least 10%; this estimate depends on the type of *de novo* mutation and can be higher; [74, 92]), these observations are suggestive of an additional, genetically driven mechanism (in addition to the two factors already mentioned, the normally protracted maturation of the cerebellum relative to other brain structures and the atypical learning process during the 1^st^ year of life), which could contribute to subtle alterations in surface structure of the right cerebellar cortex in ASD males.

Since FreeSurfer, at present, does not segment the cerebellum into separate lobules, and we wished to use this software in part due to its established reliability with data acquired under different scanning environments and protocols, our study was restricted to quantitative characterization of the entire right and left cerebellar hemispheres, instead of defining these sets according to cerebellar lobule segmentations. Note that lobule segmentation may have disrupted the careful volume matches between our ASD and TD cohorts, necessitating additional analyses that would need to take into consideration potential between-group differences in lobule volumes. Our conservative approach here has allowed us to achieve our major primary aim, to establish structural atypicalities in cerebellar GM in ASD, while conducting shape analyses without a major confounder—volume.

## Conclusions

Differences in cerebellar structure in those with ASD may affect the integration of neural signals across the brain and have functional consequences that may impact the efficient interaction and communication of the individual with their surroundings (both social and non-social). Our work is the first to characterize in-vivo morphology of the cerebellar cortex in a well-characterized sample of boys with and without ASD diagnoses during the middle and late childhood period of brain development. We show that in the absence of volume differences between ASD and TD male children, boys with ASD have significantly lower FD (specifically, reduced structural complexity or flatter, less regular surface features) of the right cerebellar cortex relative to controls. In addition, we found that increased structural complexity of the right cerebellar cortex in the presence of higher PIQ relative to VIQ in this sample, reveals more normative structural features of the right cerebellar cortex in ASD, that is, FD values closer to those of TD children. As such, atypical cerebellar morphology reveals initial *in-vivo* structural evidence of dissociable learning signatures in atypically developing children.

## Supporting information

S1 Fig Flowchart of the image processing and analyses pipeline. In Step 1, we convert the 3D object to a 3D binary image and then center the 3D image—by removing the blank space surrounding the object and 0-padding it in the x, y, and z directions. The 3D file is then covered with a 3D grid of differently sized r in order to compute *N*(r), detecting cubes that contain part of the 3D image. Specifically, *N*(*r*) represents the number of cubes required to fully cover the 3D object and the box size of the cube is *r*. We applied 20 randomly positions or “grid offsets” of the 3D grid; this parameter is defined by the number of positioning (offsets) of the object within the grid. In Step 2, we used the median *N*(r) value output from Step 1 and performed slope analysis, for *D*_2_ measures. The initial range of cube size r is [2 initRMax] and initRMax is 70% of the smallest dimension of the object. The final output of FD (*D*_2_) is the slope of the best-fitting regression line. This best-fitting line ranges between min r (2) and the breakpoint (chosen in a way that yields the line of best fit; please see main Methods text for additional details).
(TIFF)

S2 Fig Illustration of the cognitive profiles (VIQ and PIQ subscores) in ASD and TD participants from the initial ABIDE sample with available scores for both VIQ and PIQ, on either the WASI or via Ravens/PPVT, the two most frequent types of IQ assessment techniques in ABIDE. The top row shows that VIQ is lower in ASD relative to TD participants, and this pattern is overall consistent whether using the PPVT (left-most panel, red symbols), or the WASI (whole sample: middle panel, red symbols; and participants 18 years old and below: right-most panel, red symbols). The bottom row depicts same data organized by VIQ and PIQ for each subgroup. Note that for the WASI, ASD participants have higher PIQs relative to their VIQs (*P*<0.05). For additional details, please see **S1 Methods** text. The number of participants comprising the above panels is as follows. Ravens/PPVT sample: Ravens (i.e., PIQ): N = 22_ASD_, N = 50_TD_; PPVT (i.e., VIQ): N = 22_ASD_, N = 50_TD_; WASI-only sample: PIQ: N = 179_ASD_, N = 208_TD_; VIQ: N = 179_ASD_, N = 208_TD_; WASI-only = < 18 yrs old sub-sample: PIQ: N = 105_ASD_, N = 132_TD_; VIQ: N = 105_ASD_, N = 132_TD_.
(JPG)

S3 Fig Illustration of the uneven cognitive profiles (PIQ>VIQ) in ASD relative to TD participants in our subset. The TD cohort (N = 18) is comprised of participants for whom their PIQ score (blue diamonds) is higher than their VIQ score (red symbols) as well as those with the opposite pattern (VIQ score is higher than their PIQ). In contrast, only 2 out of 20 ASD participants have higher VIQ scores relative to their PIQ scores. The top panel shows data from all participants (N = 20_ASD_, N = 18_TD_) while bottom panel shows data from participants assessed with WASI instrument only (N = 18_ASD_, N = 12_TD_). Note that both panels show a similar pattern of PIQ>VIQ profile for ASD participants.
(JPG)

S1 Table Breakpoint values (median and range: min and max) for right cerebellar cortex. (DOCX)

S2 Table One- and two-sample Kolmogorov-Smirnov test for fractal dimension values of ASD and TD for right cerebellar cortex. (DOCX)

S3 Table Fractal dimension values (median and range: min and max) for left and right cerebellar cortex. (DOCX)

S1 Methods Supplementary methods. (DOCX)

S1 Results Supplementary results. (DOCX)
