# Supplementary figures and images for: Reduced structural complexity of the right cerebellar cortex in male children with autism spectrum disorder
Source: PLoS One. 2018 Jul 11;13(7):e0196964. doi: 10.1371/journal.pone.0196964 (PMC6040688; doi:10.1371/journal.pone.0196964)

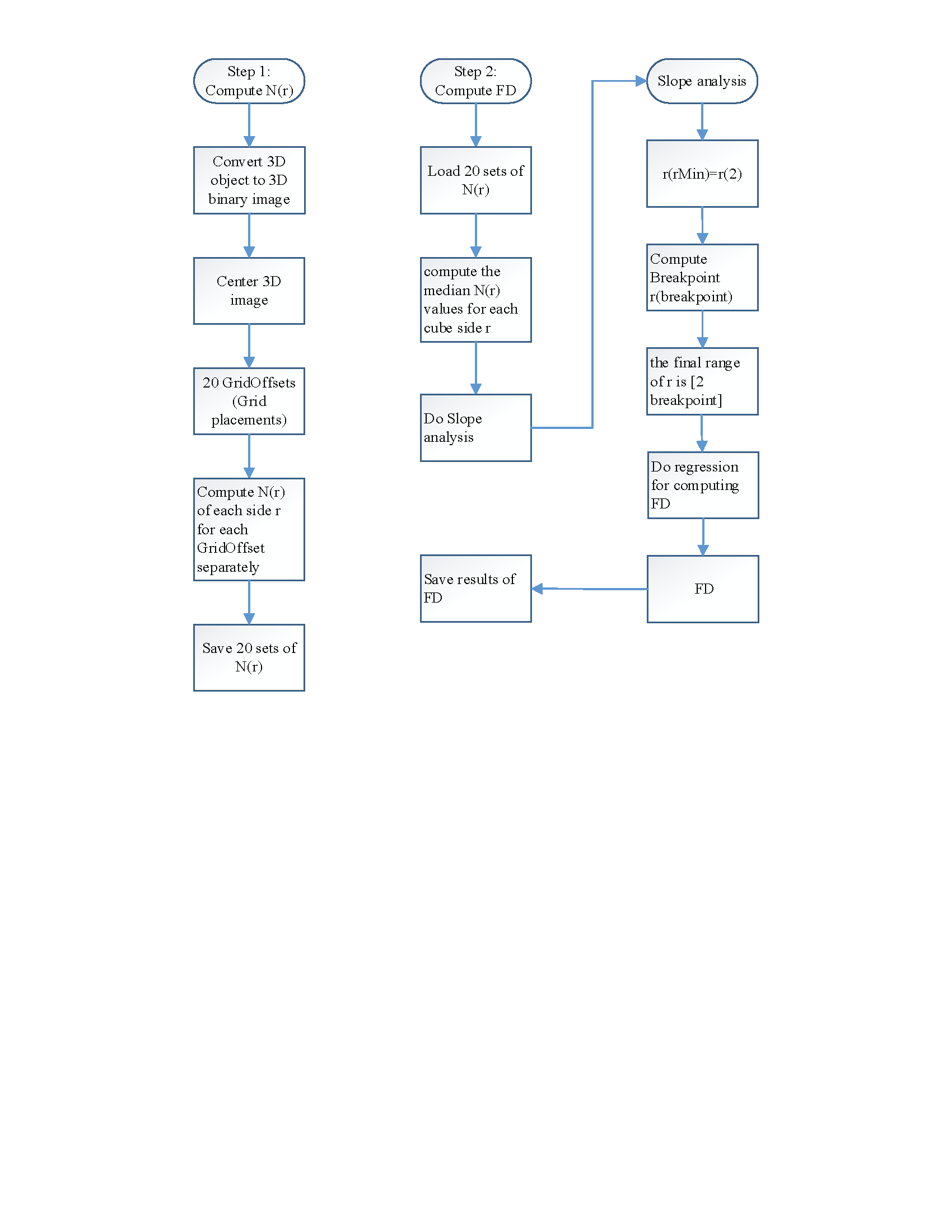

Supplement: S1 Fig — In Step 1, we convert the 3D object to a 3D binary image and then center the 3D image—by removing the blank space surrounding the object and 0-padding it in the x, y, and z directions. The 3D file is then covered with a 3D grid of differently sized r in order to compute N(r), detecting cubes that contain part of the 3D image. Specifically, N(r) represents the number of cubes required to fully cover the 3D object and the box size of the cube is r. We applied 20 randomly positions or “grid offsets” of the 3D grid; this parameter is defined by the number of positioning (offsets) of the object within the grid. In Step 2, we used the median N(r) value output from Step 1 and performed slope analysis, for D2 measures. The initial range of cube size r is [2 initRMax] and initRMax is 70% of the smallest dimension of the object. The final output of FD (D2) is the slope of the best-fitting regression line. This best-fitting line ranges between min r (2) and the breakpoint (chosen in a way that yields the line of best fit; please see main Methods text for additional details). (TIFF) [file pone.0196964.s001.tiff]

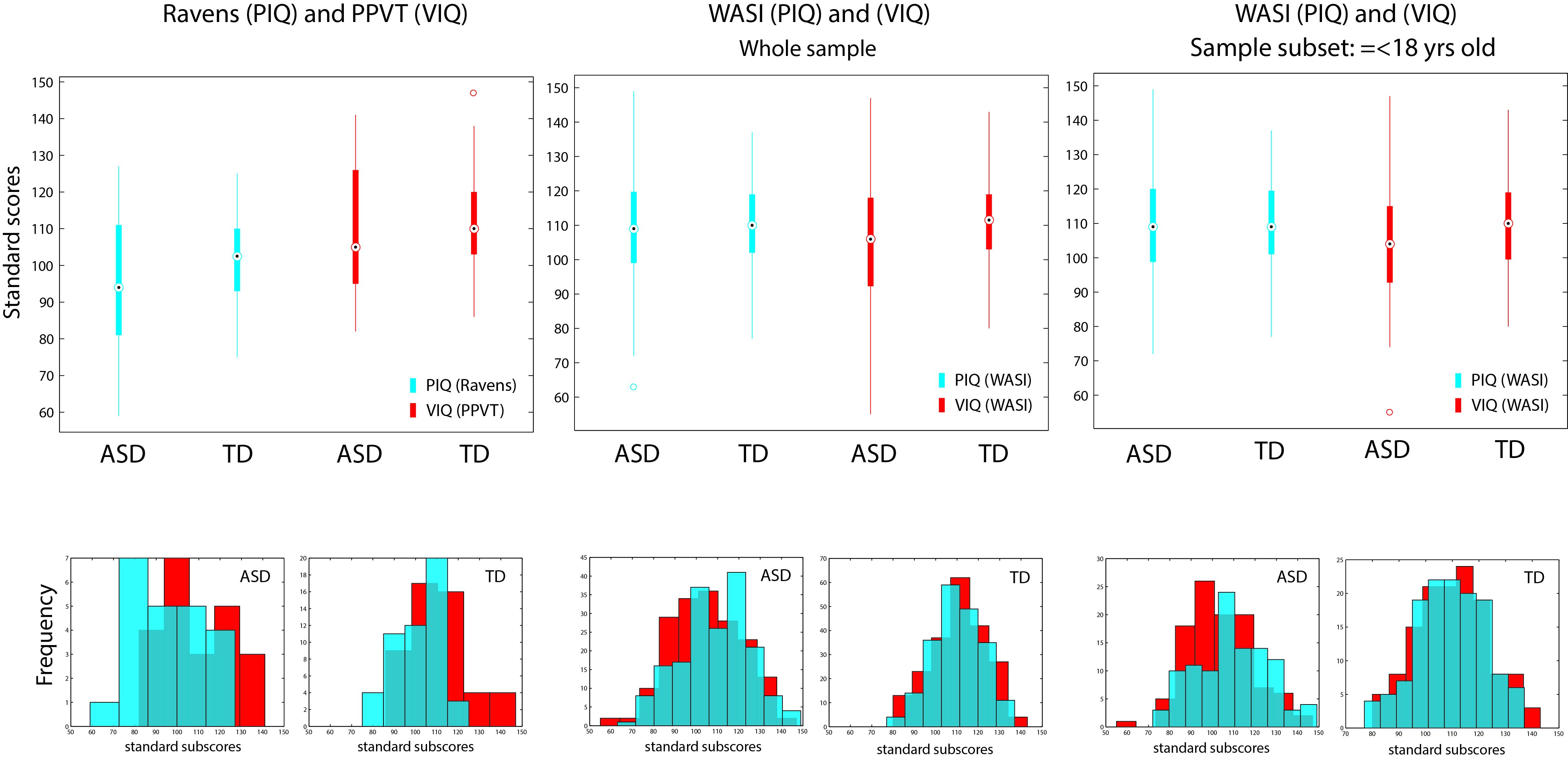

Supplement: S2 Fig — The top row shows that VIQ is lower in ASD relative to TD participants, and this pattern is overall consistent whether using the PPVT (left-most panel, red symbols), or the WASI (whole sample: middle panel, red symbols; and participants 18 years old and below: right-most panel, red symbols). The bottom row depicts same data organized by VIQ and PIQ for each subgroup. Note that for the WASI, ASD participants have higher PIQs relative to their VIQs (P<0.05). For additional details, please see S1 Methods text. The number of participants comprising the above panels is as follows. Ravens/PPVT sample: Ravens (i.e., PIQ): N = 22ASD, N = 50TD; PPVT (i.e., VIQ): N = 22ASD, N = 50TD; WASI-only sample: PIQ: N = 179ASD, N = 208TD; VIQ: N = 179ASD, N = 208TD; WASI-only = < 18 yrs old sub-sample: PIQ: N = 105ASD, N = 132TD; VIQ: N = 105ASD, N = 132TD. (JPG) [file pone.0196964.s002.jpg]

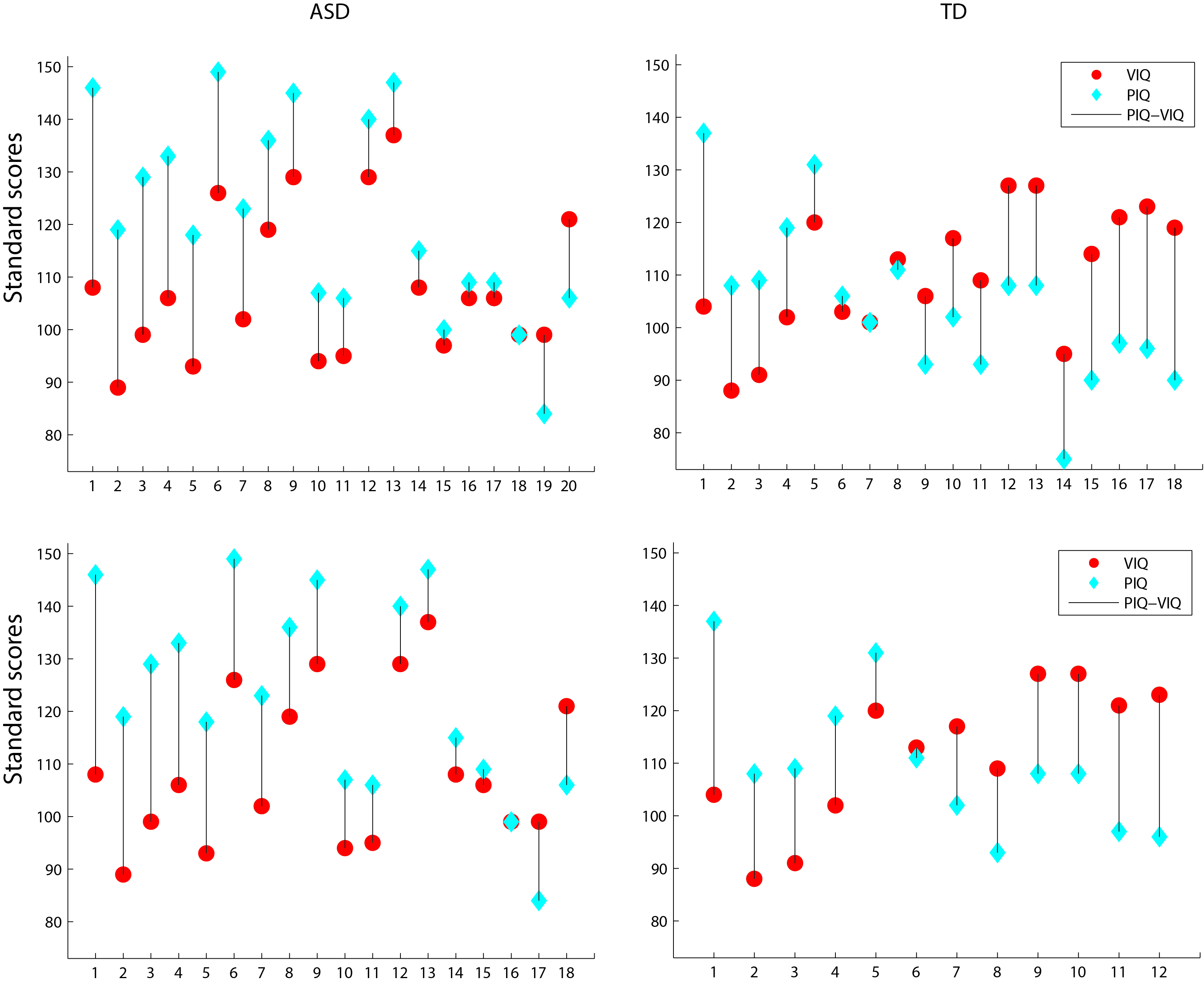

Supplement: S3 Fig — The TD cohort (N = 18) is comprised of participants for whom their PIQ score (blue diamonds) is higher than their VIQ score (red symbols) as well as those with the opposite pattern (VIQ score is higher than their PIQ). In contrast, only 2 out of 20 ASD participants have higher VIQ scores relative to their PIQ scores. The top panel shows data from all participants (N = 20ASD, N = 18TD) while bottom panel shows data from participants assessed with WASI instrument only (N = 18ASD, N = 12TD). Note that both panels show a similar pattern of PIQ>VIQ profile for ASD participants. (JPG) [file pone.0196964.s003.jpg]
