# Supplementary material for: Reduced structural complexity of the right cerebellar cortex in male children with autism spectrum disorder
Source: PLoS One. 2018 Jul 11;13(7):e0196964. doi: 10.1371/journal.pone.0196964 (PMC6040688; doi:10.1371/journal.pone.0196964)
Supplement: S1 Table — (DOCX) [file pone.0196964.s004.docx]

**Supplementary Table S1. Breakpoint values (median and range: min and max) for Right Cerebellar Cortex.**

| Structure | FD measure | Group | median | min | max | *U* | *P* |
| --- | --- | --- | --- | --- | --- | --- | --- |
| Right Cerebellar Cortex | *D*_2_ | ASD | 37 | 33 | 40 | 103.5 | 0.024^*^ |
|  |  | TD | 35 | 24 | 41 |  |  |

*Note.* ^*^ indicates that *P*<0.05.
