# Supplementary material for: Reduced structural complexity of the right cerebellar cortex in male children with autism spectrum disorder
Source: PLoS One. 2018 Jul 11;13(7):e0196964. doi: 10.1371/journal.pone.0196964 (PMC6040688; doi:10.1371/journal.pone.0196964)
Supplement: S2 Table — (DOCX) [file pone.0196964.s005.docx]

**Supplementary Table S2. One- and two-sample Kolmogorov-Smirnov test for Fractal dimension values of ASD and TD for Right Cerebellar Cortex.**

|  |  | One-sample Kolmogorov-Smirnov | | | Two-sample Kolmogorov-Smirnov | |
| --- | --- | --- | --- | --- | --- | --- |
| Structure | FD measure | Group | KSstatistic | *P* | KSstatistic | *P* |
| Right Cerebellar Cortex | *D*_2_ | ASD | 0.99408 | <0.0001^**^ | 0.48333 | 0.0151^*^ |
|  |  | TD | 0.99412 | <0.0001^**^ |  |  |

*Note.* ^*^ indicates that *P*<0.05; ^**^ indicates that *P*<0.0001.
