# Supplementary material for: Reduced structural complexity of the right cerebellar cortex in male children with autism spectrum disorder
Source: PLoS One. 2018 Jul 11;13(7):e0196964. doi: 10.1371/journal.pone.0196964 (PMC6040688; doi:10.1371/journal.pone.0196964)
Supplement: S3 Table — (DOCX) [file pone.0196964.s006.docx]

**Supplementary Table S3. Fractal dimension values (median and range: min and max) for Left and Right Cerebellar Cortex.**

| Structure | FD measure | Group | median | min | max | *U* | *P* |
| --- | --- | --- | --- | --- | --- | --- | --- |
| Right Cerebellar Cortex | *D*_2_ | ASD | 2.5511 | 2.5170 | 2.6040 | 86 | 0.006^**^ |
|  |  | TD | 2.5851 | 2.5191 | 2.6289 |  |  |
| Left Cerebellar Cortex | *D*_2_ | ASD | 2.5659 | 2.4770 | 2.6090 | 182 | 0.965 |
|  |  | TD | 2.5637 | 2.5046 | 2.6818 |  |  |

Fractal dimension (*D*_2_) structural complexity values (median and range: min and max) are shown for the left and right cerebellar cortex, for ASD and typically developing (TD) children. *Note.* ^**^ indicates that Mann-Whitney *U* test results survived Bonferroni correction*.*
