# Supplementary material for: Reduced structural complexity of the right cerebellar cortex in male children with autism spectrum disorder
Source: PLoS One. 2018 Jul 11;13(7):e0196964. doi: 10.1371/journal.pone.0196964 (PMC6040688; doi:10.1371/journal.pone.0196964)
Supplement: S1 Methods — (DOCX) [file pone.0196964.s007.docx]

**Supplementary Methods**

**Inclusion/exclusion criteria**

Because we used datasets from five different ABIDE sites, reported inclusion/exclusion criteria are similar but are not fully uniform across all sites. We note that typically developing (TD) children participants at all sites were required to be normally developing, neurologically and psychiatrically healthy individuals ascertained via a detailed health questionnaire. Both NYU and Yale (the two sites with ASD children participants used in the current work) used stringent ascertainment criteria when deciding whether to include potential participants in the ASD group, including research-reliable administration of both the ADOS and ADI-R as well as the requirement to meet DSM-IV-based clinical diagnosis of ASD (see below for details). Additional information on inclusion/exclusion criteria for each site are reported in detail at *http://fcon_1000.projects.nitrc.org/indi/abide/abide_I.html*.

*Exclusion criteria common to all sites.* Potential participants (ASD and TD) across all 5 sites were excluded if there were contraindications to MRI environment and history of head trauma with loss of consciousness. NYU excluded potential participants with chronic systemic medical conditions. Potential participants were excluded if they had any neurological disorder or history of seizures at UM_1, USM, and UCLA_1. The UCLA_1 site also excluded potential participants with tic or involuntary movement disorder, or any known genetic disorder.

*Exclusion criteria for ASD participants.* Potential ASD participants at the NYU site were excluded if they had a manic/depressive episode, bipolar disorder, schizophrenia, or posttraumatic stress disorder, as well as if they were on antipsychotic medication. Yale did not report exclusion criteria specific to potential ASD participants.

*Inclusion criteria.* Estimates of IQ (Verbal and Performance IQ) were obtained using the Wechsler Abbreviated Scales of Intelligence (WASI) [[1](#_ENREF_1)] at NYU, UCLA_1, and USM. At USM, IQ estimates for one participant was obtained using the Wechsler Intelligence Scale for Children (WISC-III) [[2](#_ENREF_2)]. Differential Abilities Scale (DAS-II: School Age Edition) [[3](#_ENREF_3)] was used at Yale. At UM_1 estimates were obtained using the Ravens Standard Progressive Matrices [[4](#_ENREF_4)] and the Peabody Picture Vocabulary Test (PPVT) [[5](#_ENREF_5)] (note that full IQ estimate is given as an average of the Ravens score (PIQ) and PPVT score (VIQ)). USM required performance IQ (PIQ)>70, UM_1 required that either VIQ or PIQ scores were >=85. Participants at UCLA_1 were reported to be required to be “fully verbal”; IQ cut-off is not indicated for NYU or Yale. (As described in the main text, in the current study, we required all participants to have Full Scale IQ>70).

*IQ assessment instruments.* In the current work we have used IQ estimates in order to perform matching between ASD and TD groups, as well as to study the potential role of differences in IQ (e.g., PIQ>VIQ) on brain measures. However, it has been noted that IQ estimates may vary across different instruments, in particular for clinical populations [[6](#_ENREF_6)] and Wechsler-based instruments may produce uneven cognitive profiles [[6](#_ENREF_6), [7](#_ENREF_7)]. Therefore, we provide pertinent details on the cognitive profiles of the meta sample below and rule out the potential role of different instruments with regard to brain measures in our complementary analyses.

The WASI is the most common instrument used to assess IQ across the entire ABIDE sample. **S2 Fig.** shows scores from ASD and TD participants assessed with the WASI [[1](#_ENREF_1)] (across all ages, minimum age ~6 years old and maximum age ~56 years old: N=179_ASD_, N=208_TD_, as well as a subset of participants 18 years old and below presented separately, N=105_ASD_, N=132_TD_) or Ravens/PPVT (scores on both tests were available for individuals 19 years old and below, for a total of N=22_ASD_, N=50_TD_), the next most frequent type of assessment. Note that overall, ASD participants have lower VIQs relative to TD participants regardless of the specific instrument used. In addition, and congruent with previous work, individuals with ASD tested on the WASI had higher scores on the PIQ relative to their VIQ scores. In contrast, TD individuals’ subtest scores on WASI were more similar, a pattern that can be appreciated by examining overlapping histograms of PIQ and VIQ in the second row of **S2** **Fig.**

Specifically, for participants with available WASI scores across all ages, the scores for ASD were: VIQ median=106 while PIQ median=109, and for TD: VIQ median=111.50 while PIQ median=110. For participants < 18 years with available WASI scores, for ASD, the scores were: VIQ median=104 while PIQ median=109, and for TD: VIQ median=110 while PIQ median=109. For participants with available Ravens/PPVT scores across all ages, for ASD, the scores were: VIQ (PPVT) median=105 while PIQ (Ravens) median=94, and for TD: VIQ (PPVT) median=110 while PIQ (Ravens) median=102.5. Individuals with ASD tested on WASI have lower VIQ relative to TD individuals for the whole sample (*P*=9.2205e-05) as well as considering younger (<18 years old) sample (*P*=0.0033). Note that while the PPVT (VIQ) scores were lower for ASD participants relative to TD, the between-group difference was not significant (*P*=0.1904). Considering WASI-only subsets, we find significantly higher PIQ relative to VIQ in ASD (whole-sample: *P*=0.0338 and <18 yrs sample: *P*=0.0265), but not for TD, whose PIQ and VIQ scores were highly similar (all *P*>0.05).

The WASI was also used by the majority of participants in our neuroimaging subset of ~7-11 year-old males for which we quantified cerebellar GM structure. Importantly for our PIQ-VIQ analyses, 90% of ASD individuals were assessed with the same instrument: WASI. Specifically, in the ASD group, N=18 were assessed with WASI and N=2 with DAS. In the TD group, N=12 were assessed with WASI, N=5 with Ravens/PPVT and N=1 with WISC-III. Thus, taking into consideration these patterns (as well as previous work reporting lower IQ scores in ASD), for our structural cerebellar analyses, we (*i*) matched individuals with respect to verbal IQ and ensured that there was no difference in Full Scale IQ, (*ii*) we repeated key imaging analyses using ASD and TD participants who were ascertained with WASI only, and (*iii*), we probed PIQ>VIQ profiles in ASD participants.

*Additional inclusion/exclusion site-specific criteria for TD participants.* TD participants at NYU, UM_1, USM, UCLA_1 were required to be neurologically and psychiatrically healthy individuals. NYU required an absence of any Axis-I disorder, confirmed using the Schedule of Affective Disorders and Schizophrenia for Children-Present and Lifetime Version (KSADS-PL [[8](#_ENREF_8)]) in TD children. UM_1 excluded potential TD participants with scores > 100 on the SCQ or > 6 on the Obsessive/Compulsive Scale of the Spence Children’s Anxiety Scale (SCAS) [[9](#_ENREF_9)]. USM required that potential TD participants had no history of learning disabilities, no history of substance abuse, psychiatric disorder, and no family history of ASD in 1^st^, 2^nd^, and 3^rd^ degree relatives. UCLA_1 required that potential TD participants “could not have a first-degree relative with ASD”.

*Inclusion criteria for ASD participants.* Both NYU and Yale conducted research-reliable administration of the Autism Diagnostic Observation Schedule (ADOS-G) and the parent interview Autism Diagnostic Interview-Revised (ADI-R) to ascertain whether participants met criteria for inclusion in the ASD group. In addition, both NYU and Yale required ASD participants to meet criteria for the DSM-IV-TR diagnosis of autism, Asperger’s or Pervasive Developmental Disorder Not-Otherwise-Specified (PDD-NOS) (note that according to the most recent version of DSM-5, individuals who would have received these diagnoses now receive a diagnosis of ASD).

*Handedness assessments.* Handedness measures were based on Edinburgh Handedness Inventory [[10](#_ENREF_10)] (NYU and USM) or on self- or parental report (Yale, UM_1, and UCLA_1). Numerical scores were converted to categorical variables (Right-handed or left-handed; none of the participants in the current work were reportedly ambidextrous).

*Recruitment.* ASD and TD participants were recruited from a variety of community sources, using advertisements, or via referrals. At NYU, ASD and TD children participants were recruited from the New York Metropolitan area, through “flyers, magazine and advertisements, parent support groups, referrals from the New York University Child Study Center clinical services, as well as word of mouth”. At Yale, ASD participants were recruited through the Simons Simplex Collection as well as through the Yale Autism Clinic.

TD participants were recruited from the community “through flyers” at UM_1, via “parent support groups, youth groups, schools, social skills groups, and other organizations” at USM, and “using flyers posted in community/youth organizations and schools, radio ads, and word of mouth” at UCLA_1. (*http://fcon_1000.projects.nitrc.org/indi/abide/abide_I.html)*

*MRI acquisition parameters.* High-resolution T1-weighted sagittal slices were acquired at 3T on Tim Trio at UCLA_1, USM, and Yale and on Allegra (Siemens, Erlangen, Germany) at NYU using MPRAGE sequence, and on a Signa (GE Medical Systems, Milwaukee, WI) at UM_1 using FSPGR sequence. At the NYU Child Study Center, structural image acquisition parameters were: [TR (Repetition Time) /TE (Echo Time)=2530/3.25 ms, TI (Inversion Time)=1100 ms, 7° flip angle, 256×192 matrix, slice thickness=1.33 mm, 128 contiguous slices)]. At Yale, acquisition parameters were: [TR/TE=1230/1.73 ms, TI=624 ms, 9° flip, 256×256 matrix, slice thickness=1 mm, 176 slices]. At UCLA_1: [TR/TE=2300 /2.84 ms, TI=853 ms, 9° flip, 256×256 matrix, slice thickness=1.33 mm, 160 slices]. At USM: [TR/TE=2300/2.91 ms, TI=900 ms, 9° flip, 256×256 matrix, slice thickness=1.2 mm, 160 slices]. At UM_1, available parameters included [TE=1.8 ms, 15° flip, 256×160 matrix, slice thickness=1.2 mm, 128 slices]. Additional details on acquisition parameters are available at: http://fcon_1000.projects.nitrc.org/indi/abide/abide_I.html.

***Cerebellum examination protocol***

Initial cerebellum (volume image) masks were obtained using default PCP anatomical image workflow resources [[11](#_ENREF_11)] in FreeSurfer, denoted as “aseg” data files. Cerebellum volume images were examined on a T1-weighted image of each participant and manually corrected, when necessary. Note that FreeSurfer assigns 1 aseg file for the Cerebellum; cerebellar left and right hemispheres are designated as “8” and “47”, respectively, with separate labels for white matter (WM) and gray matter (GM). The left and right hemisphere assignment in FreeSurfer proceeds across the midline, meaning that hemisphere designations are split across the vermis; it does not assign a separate label to the vermis. In addition, FreeSurfer does not segment cerebellar lobules. Since we were examining global properties of the cerebellum, our examination scheme is considered “coarse parcellation” according to Schmahmann [[12](#_ENREF_12)] terminology. We followed an abridged version of Bogovic et al. [[13](#_ENREF_13)] protocol with important deviations in (*ii*) noted below.

We used three planes (primarily sagittal; axial, and coronal were used as well) to inspect and correct FS aseg files (i.e., automatic segmentation-produced delineation for each subject). For *i-ii*, corrections were done, when needed, in the sagittal plane, and then checked in the axial and coronal plane.

(*i*) We first examined the GM boundary of the whole cerebellum. Specifically, following Bogovic et al. [[13](#_ENREF_13)] protocol, we examined if the FS aseg file contained any dura; none of the asegs (N=40) had any dura tissues present. We then noted whether there were any gross errors in automatic segmentation between the cerebellar GM and nearby structures including the brainstem. This step considered the “outer” boundary (GM) of the cerebellum.

(*ii*) We next examined the inner boundary proximal to the WM boundary, referred to as the corpus medullare in Bogovic et al. [[13](#_ENREF_13)]. Here we again began in the sagittal view proceeding laterally away from the midline and then reviewed in the other two planes [[13](#_ENREF_13)]. Tissues were considered as belonging to WM by strictly following FS-based boundaries. The WM label extends “to the anterior boundary of the cerebellum and the brain stem” [[13](#_ENREF_13)]. We note that the major difference between the current approach, which retains the FS-automatically segmented boundary and Bogovic et al. [[13](#_ENREF_13)] protocol is that Bogovic and colleagues’ protocol considers only those tissues that are unambiguously of the deep nuclei, and not belonging to most of the thin branches that extend into cerebellar lobules [[13](#_ENREF_13)]. In our work, large branches that were automatically segmented by FS were retained. In (*ii*), few errors were identified and corrections were minimal. In this work we focused on the structural properties of the gray matter of the cerebellum, that is, the left and right cerebellar cortices.

**Supplementary References**

1. Wechsler D. Wechsler Abbreviated Scale of Intelligence. San Antonio, Texas: The Psychological Corporation; 1999.

2. Wechsler D. Wechsler intelligence scale for children, third edition. San Antonio, TX: The Psychological Corporation; 1991.

3. Elliot CD. Differential Ability Scales (DAS). San Antonio, Texas: Psychological Corporation; 1990.

4. Raven JC. Standard Progressive Matrices. Oxford, UK: Oxford Psychological Press; 2000.

5. Dunn LM, Dunn LM. Peabody Picture Vocabulary Test (3rd ed.). Circle Pines, MN: American Guidance Services; 1997.

6. Dawson M, Soulieres I, Gernsbacher MA, Mottron L. The level and nature of autistic intelligence. Psychol Sci. 2007;18(8):657-62. Epub 2007/08/08. doi: 10.1111/j.1467-9280.2007.01954.x. PubMed PMID: 17680932; PubMed Central PMCID: PMCPMC4287210.

7. Nader A-M, Jelenic P, Soulières I. Discrepancy between WISC-III and WISC-IV Cognitive Profile in Autism Spectrum: What Does It Reveal about Autistic Cognition? PloS one. 2015;10(12):e0144645. doi: 10.1371/journal.pone.0144645.

8. Kaufman J, Birmaher B, Brent D, Rao U, Flynn C, Moreci P, et al. Schedule for Affective Disorders and Schizophrenia for School-Age Children-Present and Lifetime Version (K-SADS-PL): initial reliability and validity data. Journal of the American Academy of Child and Adolescent Psychiatry. 1997;36(7):980-8. Epub 1997/07/01. doi: 10.1097/00004583-199707000-00021. PubMed PMID: 9204677.

9. Spence SH. A measure of anxiety symptoms among children. Behav Res Ther. 1998;36(5):545-66. Epub 1998/07/02. PubMed PMID: 9648330.

10. Oldfield RC. The assessment and analysis of handedness: the Edinburgh inventory. Neuropsychologia. 1971;9(1):97-113. Epub 1971/03/01. PubMed PMID: 5146491.

11. Craddock C, Benhajali Y, Chu C, Chouinard F, Evans A, Jakab A, et al. The Neuro Bureau Preprocessing Initiative: open sharing of preprocessed neuroimaging data and derivatives. Neuroinformatics, Stockholm, Sweden; 2013.

12. Schmahmann JD, Doyon J, McDonald D, Holmes C, Lavoie K, Hurwitz AS, et al. Three-dimensional MRI atlas of the human cerebellum in proportional stereotaxic space. Neuroimage. 1999;10(3 Pt 1):233-60. Epub 1999/08/25. doi: 10.1006/nimg.1999.0459. PubMed PMID: 10458940.

13. Bogovic JA, Jedynak B, Rigg R, Du A, Landman BA, Prince JL, et al. Approaching Expert Results Using a Hierarchical Cerebellum Parcellation Protocol for Multiple Inexpert Human Raters. NeuroImage. 2013;64:616-29. doi: 10.1016/j.neuroimage.2012.08.075. PubMed PMID: PMC3590024.
