# Supplementary material for: Reduced structural complexity of the right cerebellar cortex in male children with autism spectrum disorder
Source: PLoS One. 2018 Jul 11;13(7):e0196964. doi: 10.1371/journal.pone.0196964 (PMC6040688; doi:10.1371/journal.pone.0196964)
Supplement: S1 Results — (DOCX) [file pone.0196964.s008.docx]

**Supplementary Results**

***Analyses for participants assessed with WASI***

In order to rule out the possible influence of subjects who were assessed with different IQ instruments other than the WASI on our main finding of FD reduction in ASD relative to TD participants, we repeated the main analyses (two-tailed) on data from participants who were assessed only with the WASI (N=18_ASD_, N=12_TD_). This validation analysis confirmed our main findings of reduced FD (as well as increased RMSE) in the right GM cerebellar cortex in ASD relative to TD individuals, as follows.

Specifically, a Mann-Whitney *U* test indicated a significant reduction in FD in the right cerebellar cortex in ASD relative to TD individuals (ASD median: 2.5551, range: 2.5170 - 2.6040 vs. TD median: 2.5922, range: 2.5191 - 2.6148, *U*=221, *P*=0.0149; Bonferroni corrected) (Cohen’s U3 effect size of the FD difference between groups was large, indicating a minimal overlap between FD values of ASD and TD children for the right cerebellar GM (*D*_2_, U3=0.94 (95% CIs: 0.56, 1)). Further, although highly linear, the final regression fits, *R*^2^, showed a reduction in linearity in individuals with ASD (mean +/- std): ASD, 0.9969±0.000557 and TD, 0.9976±0.000718, *P*=0.0069. In addition, we detected lower root mean square error (RMSE) values in the TD group, indicating a better fit for TD children. The RMSE values (mean +/- std) were, for the right cerebellum cortex, *D*_2_: ASD: 0.1075±0.0097, TD: 0.0946±0.00137, *P*=0.0052.

***Analyses for right-handed participants***

ASD and TD individuals did not differ in the proportion of right-handed vs. left-handed individuals (**Table 1**), and we ascertained that the main findings hold when analyzing data obtained only from right-handed individuals (N=17_ASD_, N=15_TD_). A Mann-Whitney *U* test indicated a significant reduction in FD in the right cerebellar cortex in ASD relative to TD individuals (ASD median: 2.5498, range: 2.5170- 2.5909 vs. TD median: 2.5791, range: 2.5191- 2.6289, *U*=58, *P*=0.008). The RMSE values were lower in the TD group, indicating a better fit for TD children (the RMSE values (mean +/- std) were, for the right cerebellum cortex, *D*_2_: ASD: 0.1075± 0.0086, TD: 0.0944± 0.0154, *P*= 0.0051).

***Age (and site and IQ) by diagnosis interaction***

We tested for potential interactions between age and group diagnostic status, site and diagnostic status, as well as IQ (full-scale IQ (FIQ), performance IQ (PIQ), and verbal IQ (VIQ)) and diagnostic status using linear regression. Analyses were two-tailed and included all N=38 participants (N=20_ASD_; N=18_TD_). The model included FD as the dependent variable and group, age (or site, or FIQ, or PIQ, or VIQ), and group*age (or group*site, or group*FIQ, or group*PIQ or group* VIQ) as predictor terms: Ŷ=β_0_+ β_1_(group)+ β_2_(age)+ β_3_(group*age), or Ŷ=β_0_+ β_1_(group)+ β_2_(site)+ β_3_(group*site), or Ŷ=β_0_+ β_1_(group)+ β_2_(FIQ)+ β_3_(group*FIQ), or Ŷ=β_0_+ β_1_(group)+ β_2_(PIQ)+ β_3_(group*PIQ), or Ŷ=β_0_+ β_1_(group)+ β_2_(VIQ)+ β_3_(group*VIQ). We also ran another model including RMSE (root mean square error to the log-log fits) as the dependent variable.

No age-by-diagnosis interaction was detected with FD as the dependent variable (*P*=0.439). No age-by-diagnosis interaction was detected with RMSE as the dependent variable (*P*=0.468). No site-by-diagnosis interaction was detected when considering FD (*P*=0.559) or RMSE (*P*=0.834).

No IQ-by-diagnosis interaction was detected. Specifically, no FIQ-by-diagnosis interaction was detected when considering FD (*P*=0.750) or RMSE (*P*=0.621). When considering subscales, no PIQ-by-diagnosis interaction was detected when considering FD (*P*=0.680) or RMSE (*P*=0.980). No VIQ-by-diagnosis interaction was detected when considering FD (*P*=0.942) or RMSE (*P*=0.760).

We also confirmed that there was no correlation (Spearmans’ *rho*) between FD and FIQ (*P*=0.963) or between RMSE and FIQ (*P*=0.976) when disregarding diagnostic status. There was no correlation between FD and PIQ (*P*=0.814) or between RMSE and PIQ (*P*=0.5) and no correlation between FD and VIQ (*P*=0.89) or between RMSE and VIQ (*P*=0.5).

***Relation between FD and ASD clinical outcomes and presence or absence of comorbidities***

We tested for potential association between available clinical measures on the ADOS (the social affect (SOC), repetitive and restrictive interests (RRB) scores, the Total score (TOT) comprising both scores, as well as Gotham’s Severity (SEV) score). All score values were normalized by the corresponding age and PIQ>VIQ difference score of each participant because we could not assume that similar scores from participants varying on age and PIQ>VIQ profile necessarily represent similar magnitude of clinical symptoms (ASD participants were subgrouped in a data-driven manner (rank-ordered into those with lower vs. higher scores and separated into two subgroups of participants using the median score; Wilcoxon rank-sum two-tailed test)).

We detected a trend-level finding between the SOC score and FD across 19 ASD participants for whom a meaningful normalized score could be derived (1 participant had identical PIQ and VIQ scores; since difference between scores was 0, and we could not divide by 0, data from this participant was not used). ASD participants in the “lower SOC” (i.e., “better” scores) subgroup had a higher FD (median=2.5608) while those in the “higher SOC” (“worse” scores or more symptoms) subgroup had lower FD (median=2.5445) (*P*=0.0789). Further, ASD participants in the “lower SOC” (i.e., “better” scores) subgroup had lower RMSE (median=0.1028) and in the “higher SOC” (“worse” scores or more symptoms) subgroup had higher RMSE (median=0.1118) (*P*=0.1128). No other associations were detected. No associations were detected when using raw, unnormalized ADOS scores.
